# The transcription factor CmLEC1 positively regulates the seed-setting rate in hybridization breeding of chrysanthemum

**DOI:** 10.1038/s41438-021-00625-9

**Published:** 2021-08-10

**Authors:** Sujuan Xu, Ze Wu, Huizhong Hou, Jingya Zhao, Fengjiao Zhang, Renda Teng, Liping Ding, Fadi Chen, Nianjun Teng

**Affiliations:** College of Horticulture, Nanjing Agricultural University, Key Laboratory of Landscape Design, Ministry of Agriculture and Rural Affairs, 210095 Nanjing, China

**Keywords:** Plant reproduction, Plant sciences, Fertilization

## Abstract

Distant hybridization is widely used to develop crop cultivars, whereas the hybridization process of embryo abortion often severely reduces the sought-after breeding effect. The *LEAFY COTYLEDON1* (*LEC1*) gene has been extensively investigated as a central regulator of seed development, but it is far less studied in crop hybridization breeding. Here we investigated the function and regulation mechanism of CmLEC1 from *Chrysanthemum morifolium* during its seed development in chrysanthemum hybridization. *CmLEC1* encodes a nucleic protein and is specifically expressed in embryos. *CmLEC1*’s overexpression significantly promoted the seed-setting rate of the cross, while the rate was significantly decreased in the amiR-*CmLEC1* transgenic chrysanthemum. The RNA-Seq analysis of the developing hybrid embryos revealed that regulatory genes involved in seed development, namely, *CmLEA* (late embryogenesis abundant protein), *CmOLE* (oleosin), *CmSSP* (seed storage protein), and *CmEM* (embryonic protein), were upregulated in the OE (overexpressing) lines but downregulated in the amiR lines vs. wild-type lines. Future analysis demonstrated that CmLEC1 directly activated *CmLEA* expression and interacted with CmC3H, and this CmLEC1–CmC3H interaction could enhance the transactivation ability of CmLEC1 for the expression of *CmLEA*. Further, CmLEC1 was able to induce several other key genes related to embryo development. Taken together, our results show that CmLEC1 plays a positive role in the hybrid embryo development of chrysanthemum plants, which might involve activating *CmLEA*’s expression and interacting with CmC3H. This may be a new pathway in the *LEC1* regulatory network to promote seed development, one perhaps leading to a novel strategy to not only overcome embryo abortion during crop breeding but also increase the seed yield.

## Introduction

Most plant species throughout the world rely on seed production for their reproduction, and seeds are of strategic significance to agriculture, the global food supply, and humanity^1^. Embryo morphology and embryo maturation are important biological processes in the life cycle of plants. Normal seed development is critical for protecting genetic resources and sustaining crop yields. In this respect, normal embryo development is a key feature of agriculture because it directly affects the seed-setting rate and ultimately determines the productivity of crops, thereby determining food security^2,3^.

Wild relatives of crop plants are vital reservoirs of genetic variability with respect to various economic characteristics, such as resistance against disease or insect pests, tolerance to abiotic stresses, an increased biomass, grain yield, and improved quality-related characteristics. Distant hybridization between plant varieties and wild resources is an effective way to improve the biotic and abiotic tolerance of crops and it creates many new genotypes. The ensuing hybrid offspring may be capable of greater environmental adaptability and can contribute to genetic diversity. We know that many cultivated crops, such as rice, sunflower, rape, and sorghum, were acquired via hybridization with wild resources^4–6^. Yet hybridization obstacles often emerge when carrying out distant hybridization, which hinders fertilization success and the development of hybrid embryos, seriously impairing the utilization of potentially excellent germplasm resources^7–9^. Nevertheless, much remains unknown of the regulation mechanism responsible for hybrid embryo development.

As a seed develops, the embryo goes through a series of stages under transcriptional control. In Arabidopsis and other plants, some transcriptional regulators related to seed development have been found, such as *ABSCISIC ACID INSENSITIVE3* (*ABI3*), *LEC2, FUSCA3* (*FUS3*), *WRINKLED1* (*WRI*), *BABY BOOM* (*BBM*), and *LEAFY COTYLEDON1* (*LEC1*)^10–14^. In particular, LEC1 is a highly conserved member of NF-YB protein family in eukaryote, which is necessary for normal embryo development during morphogenesis and maturation of Arabidopsis^15,16^. The *lec1* mutations with loss of function lead to defects in lipid accumulation and storage protein, acquisition of drying tolerance, and inhibition of germination^17^. *ABI3*, *LEC2*, and *FUS3* are members of the plant-specific B3 transcription factor (TF) family. In addition, BBM activates the *LEC1-ABI3-FUS3-LEC2* network and induces somatic embryogenesis^10^, and WRI1 is required for seed germination and seedling establishment^11^. In Arabidopsis, *LEC1*, *LEC2*, *ABI3*, and *FUS3* genes were identified originally as loss-of-function mutations generating defects in both processes of embryo identity and seed maturation^18–20^. The analysis of the interaction between these TFs shows that *LEC1* is the central regulatory factor of seed development^21,22^. In *Brassica napus*, *LEC1* is directly activated by *LEC2*, and together they activate the expression of genes related to promoting lipid accumulation in seeds, such as *OLE1*^23,24^. Although this is important, our understanding of the gene regulatory network controlled by *LEC1* is limited, notably for late stages of seed development.

Our previous research indicated that reproductive barriers often occur in the distant hybridization of chrysanthemum plants, in which embryo abortion is the main cause resulting in a low seed-setting rate during its wide cross^25^. Chrysanthemum embryo abortion arises from a type of programmed cell death while chromosome doubling can overcome barriers to chrysanthemums’ cross. The abundance of the CmLEC1 protein was increased during the chromosome doubling of males, which may contribute to normal embryo development. What is more, the expression level of *CmLEC1* was significantly higher in normal chrysanthemum embryos than abortive embryos, and the expression level in heart-shaped embryos surpassed that in spherical embryos^26,27^. Those findings suggest CmLEC1 may play a pivotal role in chrysanthemum embryo development. Although LEC1 has become a research “hotspot” in fundamental studies of plant embryo development, its functioning and regulatory mechanism are less explored and reported on in the context of crop hybridization breeding.

In this study, an embryo development gene, *CmLEC1*, was isolated from *Chrysanthemum morifolium* ‘Yuhualuoying’. To elucidate its functional roles, we generated transgenic lines overexpressing *CmLEC1* (OE-*CmLEC1*) and amiR plants (amiR*-CmLEC1*) in which *CmLEC1* was specifically silenced. Functional analyses revealed that CmLEC1 positively regulates seed development by activating the expression of *CmLEA*. Subsequent experiments demonstrated that CmLEC1 forms a complex with CmC3H to promote *CmLEA*’s expression and normal embryo development.

## Results

### CmLEC1 is a LEC1 homolog from chrysanthemum

**Fig. 1:**
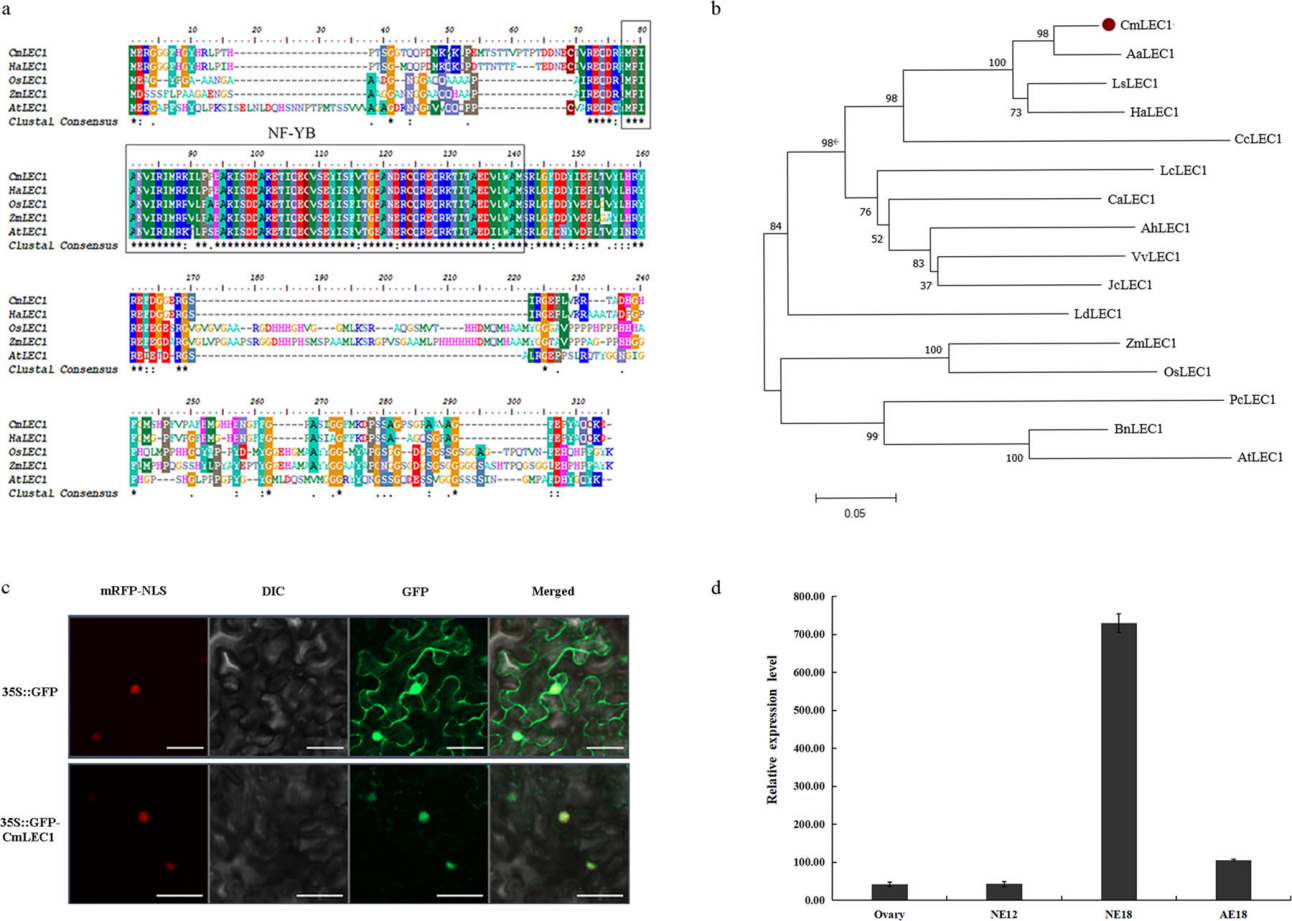
Isolation and sequence analysis of the *CmLEC1* gene. **a** Amino acid sequence alignment of CmLEC1 and plant LEC1 proteins, whose sequence features include a NF-YB/HAP3 domain. **b** Phylogenetic analysis of plants’ amino acid sequences of LEC1. **c** CmLEC1 is localized to the nucleus, based on transient expression profiles of CmLEC1 in *N. benthamiana* leaves. The co-expressed 35S::D53-RFP construct indicated the localization of nuclei. Scale bars = 5 μm. **d** *CmLEC1* is specifically expressed in chrysanthemum embryos. NE12 normal embryos at 12 days after pollination, NE18 normal embryos at 18 days after pollination, AE18 abnormal embryos at 18 days after pollination. Error bars represent ±SD

The full-length cDNA of *CmLEC1* (*CL4474.Contig1*) from chrysanthemum ‘Yuhualuoying’ is 1002 bp in size, with a 660-bp open reading frame (ORF) encoding a putative protein of 219 amino acids. CmLEC1 contained a typical NF-YB domain, and the sequence identity in common between CmLEC1 and other LEC1 homologs ranged from 39.33 to 46.91%. For example, CmLEC1 shared a 46.91% identity with AtLEC1 from Arabidopsis, 46.03% with HaLEC1 from *Helianthus annuus*, and 40.43% with OsLEC1 from *Oryza sativa* (Fig. 1a). Phylogenetic analysis confirmed that CmLEC1 is most closely related to AaLEC1 from *Artemisia annua* (Fig. 1b); the phylogenetic analysis of CmLEC1 with Arabidopsis NF-YB family revealed CmLEC1 to be clustered with Arabidopsis NF-YB6 and NF-YB9 (Fig. S1). These results confirmed that the sequence isolated from chrysanthemum was a LEC1 ortholog, thus designated here as CmLEC1.

### *CmLEC1* encodes a nucleic protein and is specifically expressed in embryos

To determine the subcellular location of CmLEC1, the transient expression of GFP-CmLEC1 fusion proteins in the *Nicotiana benthamiana* leaves was examined. The 35S::GFP-CmLEC1 signal was mainly detected in the nucleus (Fig. 1c), which indicated that CmLEC1 was a nucleus-located protein and so it might function as a TF.

The *CmLEC1* gene was expressed differently in different organs of chrysanthemum, in that the *CmLEC1* mRNA transcripts were detected primarily in ovaries and embryos but hard to detect in other plant parts. An increase in *CmLEC1*’s mRNA abundance was observed during normal chrysanthemum embryo development. Its expression level in NE18 (normal embryo, 18 days after pollination [DAP]) was significantly higher than that of NE12 (normal embryo, 12 DAP), but this augmented expression was largely absent in AE18 (aborted embryo, 18 DAP). These results revealed that *CmLEC1* is specifically expressed in both ovaries and normal embryos (Fig. 1d), suggesting that it might positively regulate the seed-setting rate during normal embryo development in chrysanthemum plants.

### CmLEC1 promotes embryo development and facilitates seed setting

**Fig. 2:**
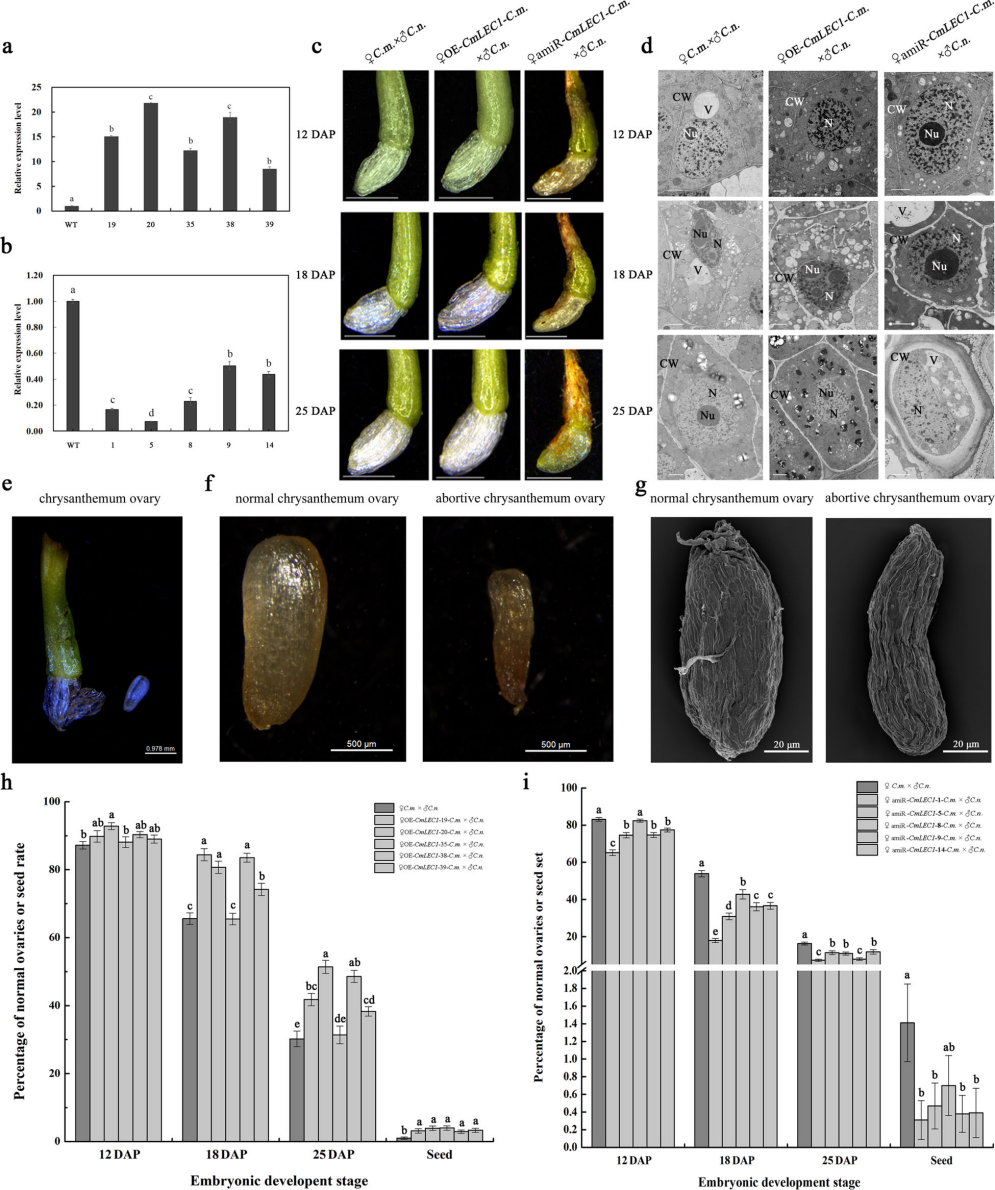
Phenotype analysis of OE-*CmLEC1* and amiR-*CmLEC1* chrysanthemum transgenic lines. **a** Relative expression level of *CmLEC1* in OE-*CmLEC1* plants. **b** Relative expression level of *CmLEC1* in amiR-*CmLEC1* plants. **c** Morphological features of the NE12, NE18, and NE25 in chrysanthemum ovaries of ♀OE-*CmLEC1*-*C.m*.×♂*C.n*. and ♀amiR-*CmLEC1*-*C.m*.×♂*C.n*. crosses. Scale bars = 1 mm. 12 DAP 12 days after pollination, 18 DAP 18 days after pollination, 25 DAP 25 days after pollination. **d** Transmission electron microscopy of NE12, NE18, and NE25 in chrysanthemum embryos of the ♀OE-*CmLEC1*-*C.m*.×♂*C.n*. and ♀amiR-*CmLEC1*-*C.m*.×♂*C.n*. crosses. Scale bar = 2 μm. Nu nucleolus, V vacuole, M mitochondria, N nucleus, ER endoplasmic reticulum, P plastid, CW cell wall. **e** Anatomical view of a chrysanthemum ovary. Scale bar = 0.978 mm. **f** Anatomical features of normal and abortive chrysanthemum ovaries. Scale bar = 500 μm. **g** Morphological characteristics of chrysanthemum ovaries. Scale bar = 20 μm. **h** Proportion of normal ovaries at different times after pollination in the ♀OE-*CmLEC1*-*C.m*.×♂*C.n*. cross. **i** Proportion of normal ovaries at different times after pollination in the ♀amiR-*CmLEC1*-*C.m*.×♂*C.n*. cross

To investigate the functions of CmLEC1, we generated OE-*CmLEC1* and amiR-*CmLEC1* lines under control of the CaMV 35S promoter (Fig. S2). Sixteen transgenic lines in which *CmLEC1* was overexpressed (OE-*CmLEC1*), and another eight in which *CmLEC1* was specifically interfered with using an artificial microRNA (amiR-*CmLEC1*), were obtained (Fig. 2a, b). The overexpressing transgenic lines were verified by PCR amplification with 35S forward primer and reverse *CmLEC1* gene-specific primer (Fig. S3a), while the amiR-*CmLEC1* transgenic lines were verified by PCR amplification using a 35S forward primer and a reverse II primer (Fig. S4a). The *CmLEC1* expression levels in these transgenic lines were validated by quantitative reverse transcription PCR (qRT-PCR; Figs. S3b and S4b). From both sets of transgenic constructs, five independent lines were selected for use in further experiments (Fig. 2a, b).

To assess the effects of *CmLEC1* overexpression or knockdown upon the seed set of hybridized plants, the tetraploid *Chrysanthemum nankingense*’s pollen was pollinated onto the *C. morifolium* stigmas of the wild-type (WT), OE-*CmLEC1*, and amiR-*CmLEC1* plants (Fig. S5). The hybrid embryos at 12 DAP, 18 DAP, and 25 DAP of non-transgenic plants and transgenic plants were initially examined under morphological microscopy and transmission electron microscopy (TEM): the embryo morphologies were highly correlated with *CmLEC1*’s expression level. The ovaries of the ♀OE-*CmLEC1*-*C.m*.×♂*C.n*. cross was relatively full until the seeds formed, while those of the ♀amiR-*CmLEC1*-*C.m*.×♂*C.n*. cross were relatively small and hollow, when compared with those of the ♀*C.m*.×♂*C.n*. cross (Fig. 2c). TEM examinations uncovered no significant differences among the ♀*C.m*.×♂*C.n*., the ♀OE-*CmLEC1*-*C.m*.×♂*C.n*., and ♀amiR-*CmLEC1*-*C.m*.×♂*C.n*. crosses at 12 DAP. For each, the nucleolus could be clearly observed. Some typical cell organelles, such as mitochondria, the Golgi complex, and vacuoles or plastids with normal shapes were also clearly apparent. In addition, the cells have complete cell wall structure and rich abundant formation. At 18 DAP, compared with the ♀*C.m*.×♂*C.n*. embryos, in the ♀OE-*CmLEC1*-*C.m*.×♂*C.n*. embryonic cells, though their cytoplasm had shrinked slightly during embryonic development, their organelles developed well and metabolism in mitochondria was very active and they had accumulated more fat. By contrast, at 18 DAP, embryonic cells of the ♀amiR-*CmLEC1*-*C.m*.×♂*C.n*. cross looked significantly different, with an evident shrinkage of nuclei along with degraded organelles, plasma wall separation, and no fat accumulation. At 25 DAP, there was more protein and starch in the ♀OE-*CmLEC1*-*C.m*.×♂*C.n* cells compared with ♀*C.m*.×♂*C.n*. cells, whereas neither could be observed in the ♀amiR-*CmLEC1*-*C.m*.×♂*C.n*. embryonic cells whose organelles had gradually degraded and whose cell walls appeared distorted and thickened (Fig. 2d). These results suggested that CmLEC1 was involved in the regulation of chrysanthemum embryo development.

To further assess the role of CmLEC1 during hybrid embryo development, the ratio of normal ovaries arising from different pollinations at differing developmental stages was derived (Fig. 2e). First, the morphological characteristics of ovules were observed under a morphological microscope and scanning electron microscope. The normal ovaries appear on the left and abnormal ones on the right of Fig. 2f, g, and these were used as the standard for the statistical analysis. At 12 DAP, the globular embryo structure could be observed (Fig. S6a, b) and ~87.2% of the ♀*C.m*.×♂*C.n*. ovaries completely developed, indicating normal embryo development. The ♀OE-*CmLEC1*-*C.m*.×♂*C.n*. cross had a greater percentage of normal ovaries (Fig. 2h). By contrast, in most cases of the ♀amiR-*CmLEC1*-*C.m*.×♂*C.n*. cross, except that of ♀amiR-*CmLEC1*-*C.m.-*8×♂*C.n*., reductions in the percentage of normal ovaries were evident (Fig. 2i). At 18 DAP, for the ♀*C.m*.×♂*C.n*. cross more shriveled ovaries were observed, indicative of abnormal embryo development. Hence, there was a significant decrease (of ~29.2%) in the percentage of morphologically normal ovaries going from 12 DAP to 18 DAP; however, this reduction was significantly alleviated in the ♀OE-*CmLEC1*-*C.m*.×♂*C.n*. cross. Except in the case of ♀OE-*CmLEC1*-*C.m.-*35×♂*C.n*., every other ♀OE-*CmLEC1*-*C.m*.×♂*C.n*. cross exhibited significantly higher ratios of normal ovaries in comparison with the ♀*C.m*.×♂*C.n*. cross at 18 DAP. In contrast, each ♀amiR-*CmLEC1*-*C.m*.×♂*C.n*. cross was distinguished by a significant reduction in the percentage of normal ovaries (up to 36%). At 25 DAP, the cotyledon embryo structure could be observed (Fig. S6c, d), and most of the ovaries were shriveled and the proportion of normal ovaries declined further, to ~30.2% for the ♀*C.m*.×♂*C.n*. cross. Yet a significant increase, of up to 21.2%, occurred in the proportion of normal ovaries for ♀OE-*CmLEC1*-*C.m*.×♂*C.n*., whereas the ♀amiR-*CmLEC1*-*C.m*.×♂*C.n*. cross displayed a significant reduction in the normal ovary ratio. Together, these results demonstrated that CmLEC1 might be a positive regulator of hybrid embryo development in chrysanthemums.

During normal hybridization in the ♀*C.m*.×♂*C.n*. cross, the seed-setting rate was ~1.02% but this was much higher, at ~4.0%, in the ♀OE-*CmLEC1*-*C.m*.×♂*C.n*. cross, corresponding to a significant 3.92-fold increase. Conversely, the seed-setting rate of the ♀amiR-*CmLEC1*-*C.m*.×♂*C.n*. cross was significantly reduced, by ~78.0%, relative to that of the ♀*C.m*.×♂*C.n*. cross. These results indicated that CmLEC1 regulates embryo development and facilitates the seed-setting rate in chrysanthemum hybridization breeding.

### CmLEC1 regulates the expression of seed development-related genes

**Fig. 3:**
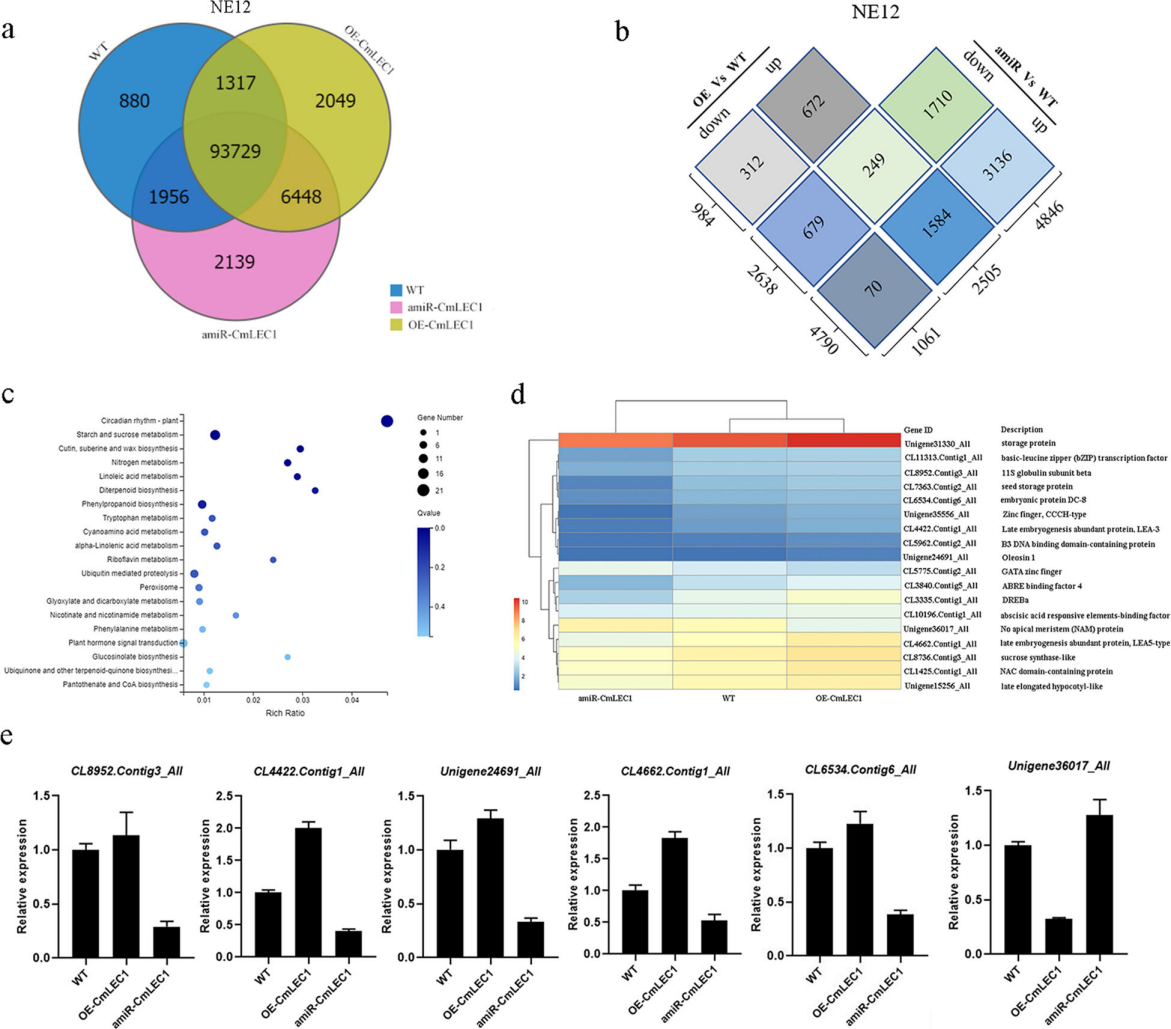
RNA-Seq analysis of plants with an altered CmLEC1. **a** Venn diagram of the number of genes in OE, amiR, and WT (wild-type) plants obtained by RNA-Seq. **b** Number of differentially expressed genes (DEGs) between OE, amiR, and WT obtained by RNA-Seq. **c** KEGG analysis; on the *x*-axis is the enrichment ratio, and on the *y*-axis are the KEGG pathways, for which a bubble’s size indicates the number of genes annotated to a certain KEGG pathway. **d** Heat map of the DEGs based on the RNA-Seq analysis of OE and amiR. The color scale indicates the scale of each gene expression level (log2FPKM). Rectangles in red denote genes’ upregulation, those in blue their downregulation. **e** Identification of the genes from DEGs related to seed development by qRT-PCR. The values are presented as the mean ± SE (*n* = 3)

To better understand how CmLEC1 mediated the seed development process, we performed an RNA sequencing (RNA-Seq) analysis of the developing hybrid embryos—these obtained from the previous experiment—where the tetraploid *C. nankingense*’s pollen was pollinated onto the *C. morifolium* stigmas of the WT, OE-*CmLEC1*, and amiR-*CmLEC1* plants. After filtering out any low-quality reads, each genotype has about 70 million clean reads (Supplemental Table S1-1). Further comparisons of the RNA-Seq data among the OE, amiR, and WT plants (*Q*-value < 0.001) revealed 8412 differentially expressed genes (DEGs). Compared with the WT, 4790 upregulated and 2638 downregulated genes were found in the amiR lines, whereas 2505 upregulated and 1061 downregulated genes were found in the OE lines (Fig. 3a, b). In addition, 249 genes were upregulated in the OE chrysanthemum but downregulated in the amiR chrysanthemum, and 70 genes were downregulated in the OE chrysanthemum but upregulated in the amiR chrysanthemum. Therefore, 319 genes were regulated in an opposite manner in the OE and amiR lines of *CmLEC1* (Fig. 3b).

We functionally annotated and classified the 249 genes using the Kyoto Encyclopedia of Genes and Genomes (KEGG) database. The KEGG analysis revealed these DEGs to be enriched in such starch and sucrose metabolism (ko00500), plant hormone signal transduction (ko04075), phenylpropanoid biosynthesis (ko00940), and key processes such as plant circadian rhythm (ko04712) (Fig. 3c). BinGO (Gene Ontology) functional analysis of the 249 genes that were upregulated in the OE lines but downregulated in the amiR line revealed that the most abundant classified term was “response to chemical stimulus”, “organic acid biosynthetic process”, “two-component signal transduction system (phosphorelay)”, and “signaling process” (Fig. S7). And the 70 genes that were downregulated in the OE lines but upregulated in the amiR line revealed that the most abundant classified term was “oxidoreductase activity”, “cofactor binding”, “NAD or NADH binding”, and “coenzyme binding” (Fig. S8). Among the 249 genes upregulated in the OE lines but downregulated in the amiR lines vs. the WT lines were several regulatory genes involved in seed development, namely *CmLEA* (*CL4422.Contig1_All*), *CmOLE* (*Unigene24691_All*), *CmSSP* (*CL5363.Contig2_All*), and *CmEM* (*CL6534.Contig6_All*). Among the 70 genes found upregulated in the amiR lines but downregulated in the OE lines vs. the WT lines were two TFs, NAM (*Unigene36017_All*) and the GATA (*CL5775.Contig2_All*) zinc finger (Fig. 3d). In order to further validate the expression profiles acquired from the transcriptome data, several genes related to seed development were selected and their transcription was detected by qRT-PCR. As seen in Fig. 3e, the results of qRT-PCR were consistent with the RNA-Seq data. Collectively, the expression results above confirmed that RNA-Seq was reliable and that CmLEC1 regulates embryo development by influencing the expression of those key genes involved in the synthesis and storage of proteins and oils.

### CmC3H directly interacts with CmLEC1

To identify the possible interacting partners of CmLEC1, the RNAs were extracted from chrysanthemum embryos and used to build a yeast two-hybrid (Y2H) library (Figs. S9 and S10). Then a Y2H screen was carried out utilizing the embryonic cDNA library of chrysanthemum ‘Yuhualuoying’ (Fig. S11). Through the screening of 1.2 × 10^7^ recombinant cDNA clones, 11 positive colonies were obtained (Supplemental Table S1-2). One of these was identified as a member of the CCCH TFs, which reportedly participated in many aspects of plant growth, development, and defense^28^. Hence, we named this gene *CmC3H*.

**Fig. 4:**
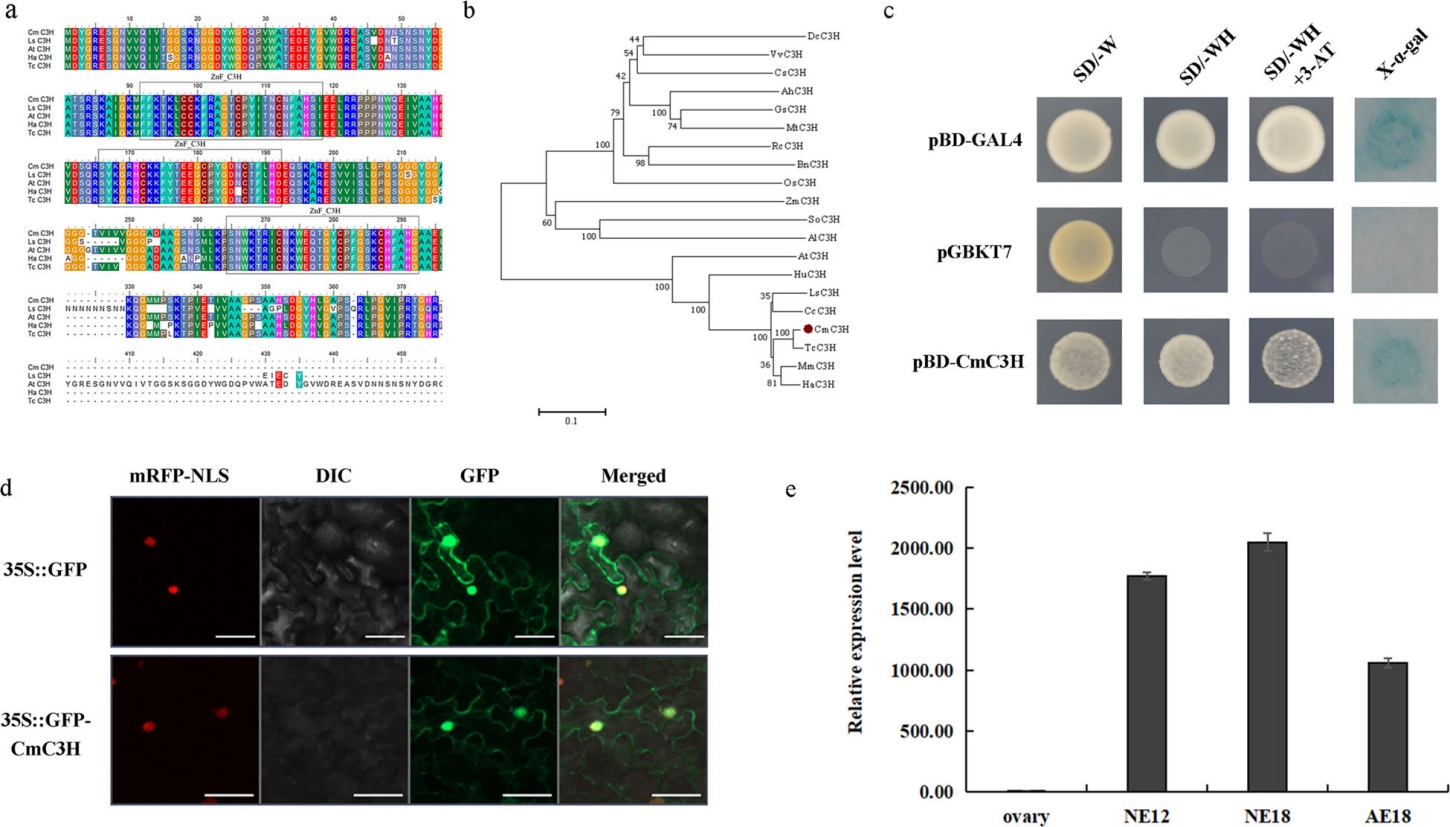
Isolation and sequence analysis of the *CmC3H* gene. **a** Amino acid sequence alignment of CmC3H and plant C3H proteins, whose sequence features include three ZnF_C3H domains. **b** Phylogenetic analysis of plants’ amino acid sequences of C3H. **c** Transcriptional activation assay of CmC3H. The selected clones were placed onto SD/-Trp-His medium and cultured at 30 °C for 2–3 days, prior to using them in the assay of X-α-galactosidase activity; pGBKT7 was used as negative control and pBD-GAL4 was used as positive control; SD/-W: SD/-Trp; SD/-WH: SD/-Trp-His. **d** Subcellular location of CmC3H in *N. benthamiana* leaves. The tobacco leaf cells transfected with 35S::GFP-CmC3H and 35S::D53-RFP were observed by confocal microscope. The nuclear marker was the co-expressed 35S::D53-RFP construct. Scale bars = 5 μm. **e** Expression analysis of *CmC3H* in different tissues of chrysanthemum ‘Yuhualuoying’. Error bars represent ±SD

The *CmC3H* gene consisted of a 1155-bp ORF, encoding a protein with 384 aa that contained three typical ZnF_C3H domains. Comparing the CmC3H protein sequence with that of other C3H proteins revealed a sequence identity that ranged from 48.96 to 97.40%. In this respect, CmC3H shared 48.96, 67.47, 90.13, and 97.40% of its identity with the C3Hs from *Artemisia annnua*, *Lactuca sativa*, *H. annuus*, and *Tanacetum cinerariifolium*, respectively (Fig. 4a). These results confirmed the CmC3H isolated from chrysanthemum was in fact a C3H homolog. Phylogenetic analysis established that CmC3H is most closely related to the AaC3H from *A. annua* (Fig. 4b). Corroborating this view, the phylogenetic analysis of CmC3H with the Arabidopsis CCCH family showed that CmC3H clustered with Arabidopsis AtC3H12 (At1g32360) (Fig. S12). The subcellular localization assay showed the CmC3H located in the nucleus and cytoplasm (Fig. 4c), and further analysis indicated that CmC3H exhibited transcriptional activation activity in yeast cells (Fig. 4d). Similar to CmLEC1, at 18 DAP the expression level of *CmC3H* was significantly higher in normal embryos than in abortion embryos (Fig. 4e).

**Fig. 5:**
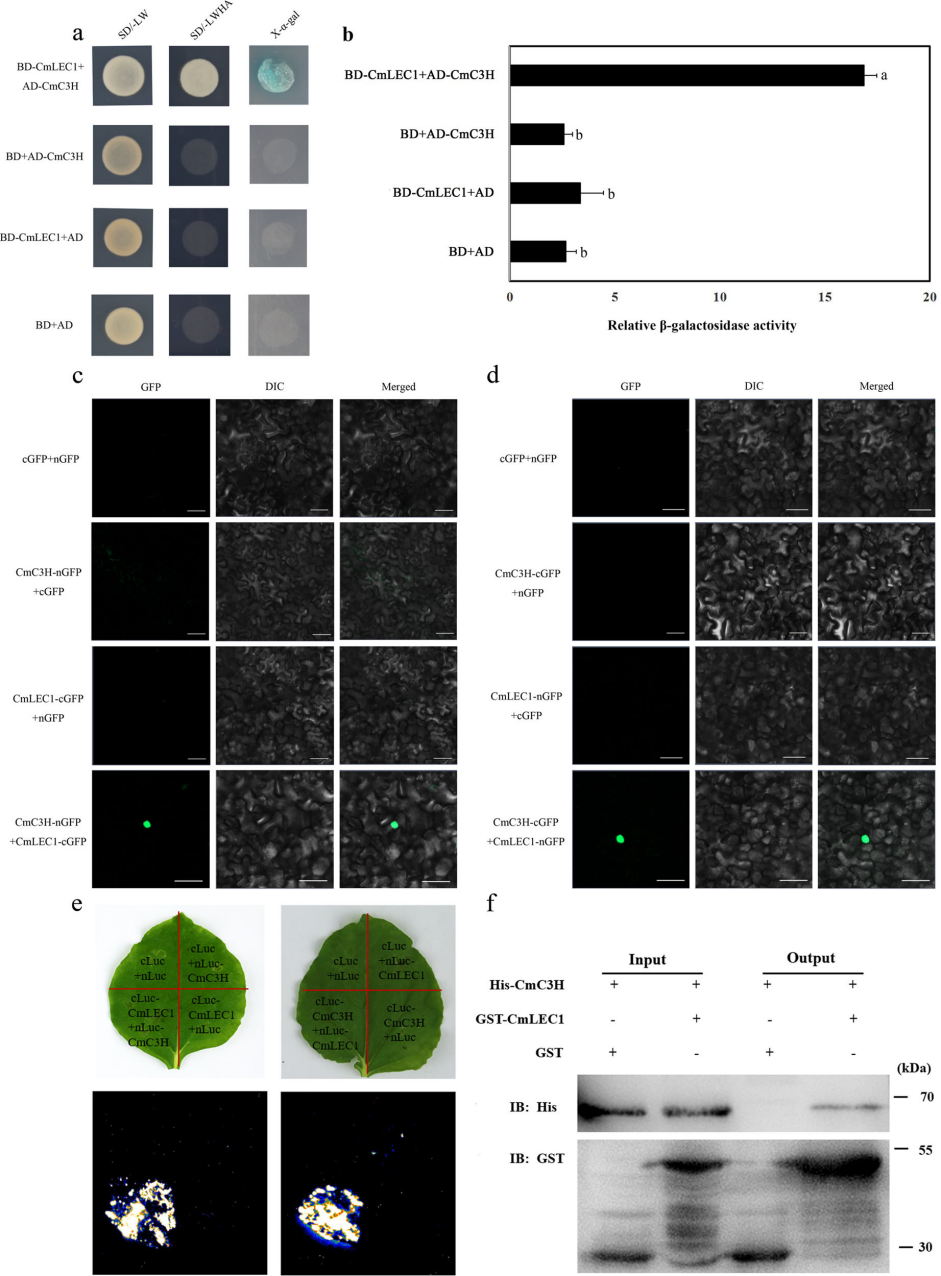
Interactions between the CmLEC1 and CmC3H proteins. **a** CmLEC1 and CmC3H; the negative control was pGADT7-T + pGBKT7, SD/-LW: SD/-Leu-Trp; SD/-LWHA: SD/-Leu-Trp-His-Ade. **b** The activity of β-galactosidase was determined by an enzyme assay. Data are the average value (±SD) of three independent experiments. Letters indicate a significant difference at *P* < 0.05, based on Student’s *t* test. **c** Interaction between CmLEC1 and CmC3H in the BiFC assays. Fluorescence was observed in the transformed cells, which resulted from complementation between the N-terminal region of GFP fused with CmLEC1 (CmLEC1-nGFP) and the C-terminal region of GFP fused with CmC3H (CmC3H-cGFP). The experiments were performed at least five times using different batches of *N. benthamiana* plants. **d** Interaction between CmLEC1 and CmC3H in the BiFC assays. Fluorescence was found in the transformed cells, which resulted from complementation between the C-terminal region of GFP fused with CmLEC1 (CmLEC1-cGFP) and the N-terminal region of GFP fused with CmC3H (CmC3H-nGFP). The experiments were performed at least five times using different batches of *N. benthamiana* plants. **e** Interaction between CmLEC1 and CmC3H in LCI assays. The LUC activity was determined 72 h later, using a CCD (charge coupled device) camera (Tanon 5200, China). **f** Interaction between CmLEC1 and CmC3H in an in vitro pull-down assay. The recombinant GST-CmLEC1 fusion was mixed with His-CmC3H fusion protein in equal volumes; following their incubation, the protein was purified by a GST column. In vitro-translated GST protein was used as a negative control. “Input” refers to the protein mixtures before the experiment; “Pull-down” means the purified protein mixture. The “+” indicates an existence, and the “−” indicates a non-existence. IB immunoblot

The interaction between CmLEC1 and CmC3H was determined by a point-to-point Y2H assay. Yeast cells co-expressing AD-CmC3H + BD-CmLEC1, but not those co-expressing pGAD + BD-CmLEC1, grew on the SD-Leu/-Ade/-His/-Trp/ screening medium (Fig. 5a). Furthermore, the β-galactosidase activity of AD-CmC3H + BD-CmLEC1 co-expressed in the transformed yeast cells significantly exceeded that of AD + BD-CmLEC1, AD-CmC3H + BD, and AD + BD (Fig. 5b), thereby confirming that CmLEC1 interacted with CmC3H in yeast cells.

To further verify that the CmLEC1–CmC3H interaction occurred in plant cells, the bimolecular fluorescence complementation (BiFC) assays were carried in *N. benthamiana* leaves. The green fluorescent protein (GFP) signals were observed in the nucleus only when CmLEC1-cGFP and CmC3H-nGFP or CmLEC1-nGFP and CmC3H-cGFP were co-expressed (Fig. 5c, d), which suggested the CmLEC1–CmC3H interaction only happened in the nucleus. A luciferase complementation imaging (LCI) assay using *N. benthamiana* leaves was also performed to detect the CmLEC1–CmC3H interaction. Strong luciferase activity signals were observed when nLUC-CmLEC1 and cLUC-CmC3H or cLUC-CmLEC1 and nLUC-CmC3H were co-expressed, whereas all the negative controls did not produce such signals (Fig. 5e). These results indicated CmLEC1 and CmC3H are co-localized and are capable of interacting in the nucleus of plant cells. Moreover, the physical interaction between CmLEC1 and CmC3H was further confirmed by a pull-down experiment in vitro. Recombinant CmC3H tagged with His (molecular weight of ~59 kDa) and recombinant CmLEC1 tagged with GST (~51 kDa) were successfully expressed and purified (Fig. S13). These results showed that both His-CmC3H and GST-CmLEC1 existed in whole-cell lysate and could be distinguished (Input). Importantly, CmC3H was not detected in the control sample (GST protein alone), but CmC3H fused to His tag was pulled down by the GST-CmLEC1 (Fig. 5f), indicating that CmLEC1 directly interacted with CmC3H.

### CmLEC1 and CmC3H synergistically activate the expression of *CmLEA*

**Fig. 6:**
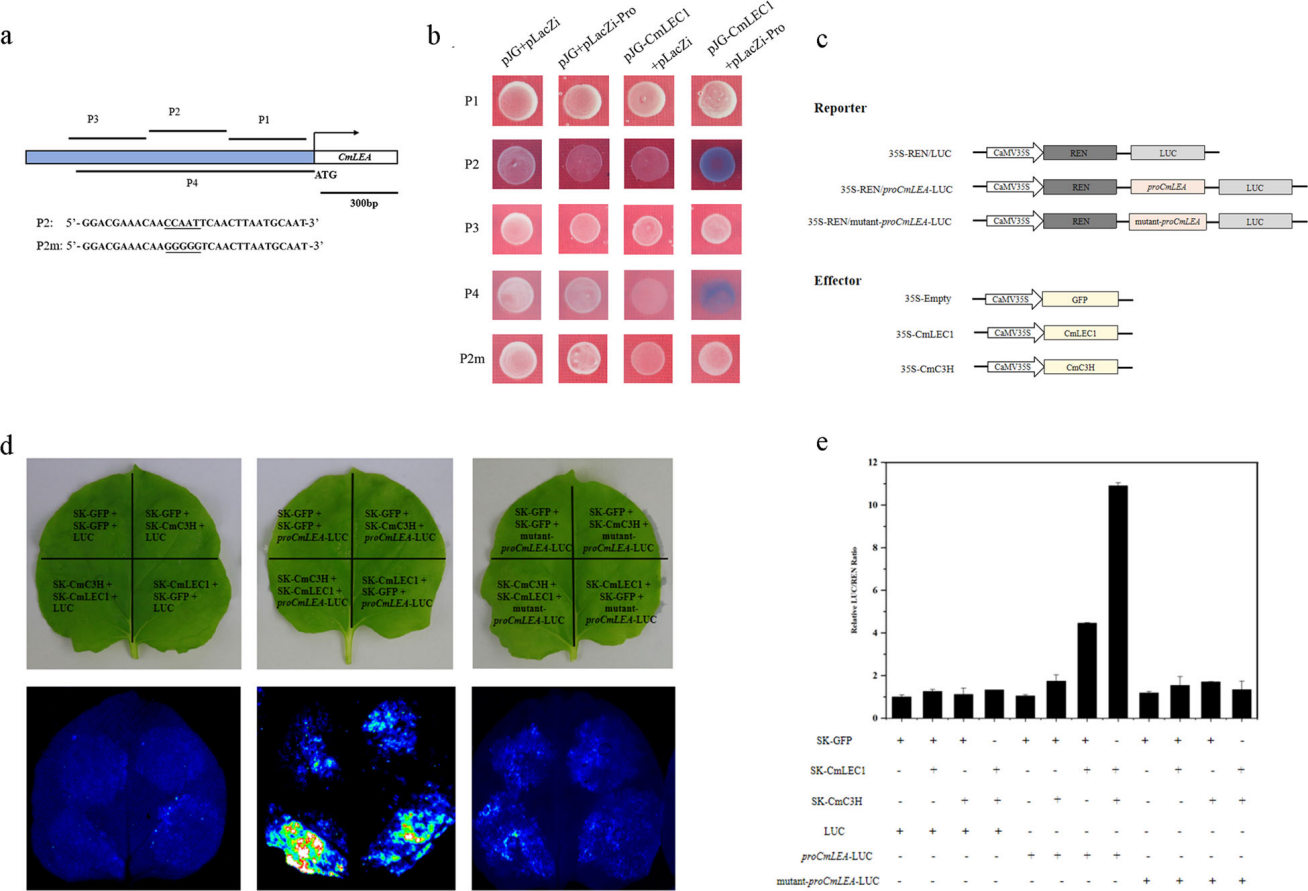
CmLEC1 and CmC3H synergistically activate the expression of *CmLEA*. **a** Schematic diagram of upstream region corresponding to *CmLEA* folded-back structure. P1: 0 to −307 bp. P2: −307 to −607 bp. P3: −607 to −907 bp. P4: 0 to −907 bp. The lines below and above the blue box were fragments used in the yeast one-hybrid assays. Nucleotide substitutions in the wild-type *cis* element (CCAAT-box) and variant (P2m) of P2 are underlined. **b** The binding of CmLEC1 and *CmLEA* promoter in the yeast one-hybrid system was analyzed. The empty vector (pJG + pLacZi) was used as negative control. The *CmLEA* promoter was ligated to the pLacZi vector in yeast cells, and the ORF of CmLEC1 was cloned into pB42AD to obtain pB42AD-CmLEC1. **c** Schematic diagram of the double-reporter and effector plasmids for dual-luciferase (LUC) reporter assay. **d**, **e** CmLEC1 and CmC3H synergistically activated the expression of *CmLEA* in *N. benthamiana* leaves. In this experiment, a 0 to −907 bp promoter fragment of *CmLEA* was used; the constructs used in the assay are as shown above. Corresponding effectors and reporters were co-infiltrated into the tobacco leaves. **d** Representative images of fluorescence signals 3 days after infiltration are shown. **e** Relative LUC/REN ratios given were measured. The experiments were independently repeated three times and mean value ± SD are shown from three replicates

To further investigate whether CmLEC1 could bind to the *CmLEA* promoter, we isolated a 907 bp promoter sequence upstream of the translation initiation site ATG. In the promoter region, a number of typical *cis*-acting elements were predicted, including a CCAAT-box element, which can be bound by NF-Y factors. In order to determine the binding of CmLEC1 to *CmLEA*’s promoter, CmLEC1 was used as the prey for Y1H, and the bait was generated by using the promoter fragments containing the original or mutated CCAAT-box. The Y1H assay showed that CmLEC1 is able to bind to the P2 fragment (−307 to −607 bp) of the promoter of *CmLEA* in yeast, whereas this binding activity was entirely lost via the mutation of the CCAAT-box (Fig. 6a, b). Further, the Y1H assay also demonstrated that Arabidopsis AtLEC1 could bind to the *AtLEA* promoter in yeast cells (Fig. S14). That LEC1 can bind to the *LEA* promoter in different species suggested that it might be a conserved mechanism. In addition, a dual-luciferase reporter assay was performed to analyze, in vivo, the regulation of *CmLEA* promoter activity by CmLEC1. For this, we fused the promoter sequences of *CmLEA* and a mutated *proCmLEA* to firefly luciferase (LUC), to generate two reporter constructs (*proCmLEA*-LUC and mutant-*proCmLEA-*LUC), and then used SK-CmLEC1 and SK-CmC3H as two different effectors (Fig. 6c). The *N. benthamiana* leaves co-transformed with SK-CmLEC1 and *proCmLEA*-LUC displayed significantly higher LUC activity than those transformed with SK-GFP and *proCmLEA*-LUC or SK-CmC3H and *proCmLEA*-LUC, respectively. Furthermore, when *proCmLEA-*LUC was replaced with the mutated *proCmLEA-*LUC, the LUC activity disappeared (Fig. 6d, e). Altogether, these results demonstrated CmLEC1 could bind to the promoter of the *CmLEA* gene to activate its expression in vivo.

To further study whether the CmC3H–CmLEC1 interaction affected the expression of *CmLEA*, a dual-luciferase reporter assay was performed to analyze, in vivo, the regulation of *CmLEA* promoter activity by CmLEC1 and CmC3H. After co-transforming SK-CmLEC1, SK-CmC3H, and *proCmLEA*-LUC into *N. benthamiana* leaves, we observed significantly higher LUC activity than those transformed with SK-CmLEC1 and *proCmLEA*-LUC and SK-CmC3H and *proCmLEA*-LUC, respectively (Fig. 6d, e). Taken together, these results indicated that the interaction between CmLEC1 and CmC3H could enhance the transactivation ability of CmLEC1 for the expression of *CmLEA*.

## Discussion

The successful maturation of embryos is an important step in the reproduction of higher plants. However, seeds do not always develop viable embryos. This embryonic abortion usually occurs during plant hybridization events, thus severely reducing the yields of seed and fruit of many crops while also adversely affecting the efficiency of plant hybrid breeding^1,3^. Previous studies have uncovered many genes related to plant embryo development. The *LEAFY COTYLEDON* (*LEC*) gene is among the key factors crucially involved in controlling both middle and late embryogenesis. The uniqueness of *LEC* genes lies in them being essential for normal embryo development in both the morphogenesis and maturation stages^29,30^. Ectopic expression of *LEC1* induces the activation of genes related to maturation as well as genes related to lipid and protein accumulation in vegetative organs^31^. Additionally, the *lec1* mutation entailing a loss of function could lead to pronounced plant physiological defects with respect to accruing stored protein and lipids, acquiring tolerance for desiccation, and inhibiting seed germination and leaf primordia initiation^32,33^. We found *CmLEC1* highly expressed in normal developing embryos (Fig. 1d), and its overexpression partly overcame hybrid embryo abortion, but the microRNA-mediated silencing of *CmLEC1* led to more severe embryo abortion when compared with the non-transgenic plants (Fig. 2e, f).

The development of plant seeds consists of a series of stages under strict transcriptional control. Further, various proteins are stably synthesized in the latter half of embryogenesis, such as late LEA (late embryogenesis-abundant) protein and the seed storage protein^34^. In plants, the former denotes a large class of hydrophilic proteins linked to desiccation tolerance during the process of embryo maturation^35,36^. Their genes are expressed late in embryogenesis and this *LEA* gene expression can be used as a convenient marker for changed expression patterns in dormancy mutants or in mutations that affect dormancy^37^. The developmental mutants *lec1* and *fus3* show similar changes in their levels of *LEA* gene expression. When compared with the WT, the expression levels in mature seeds of *lec1-1* and *lec1-2* at different stages of development were reduced for the AtLEA protein, in both mutants^38^. In our previous work, we had found that more *LEA* genes were found expressed at NE18 in the cross of *C. morifolium* × tetraploid *C. nankingense* than in that of *C. morifolium* × diploid *C. nankingense*^27^. Because the expression of *LEA* affects both dormancy and desiccation tolerance during embryo maturation, it could explain why we were able to acquire seeds.

Plant embryogenesis is a complex process, in which various proteins such as LEA, OLE, and SSP are stably synthesized^39–41^. In our study, the expression levels of *CmLEA*, *CmOLE*, *CmSSP*, and *CmEM* genes were all upregulated in the ♀OE-*CmLEC1*-*C.m*.×♂*C.n*. cross, yet downregulated in the ♀amiR-*CmLEC1*-*C.m*.×♂*C.n*. cross, when compared with the ♀*C.m*.×♂*C.n*. cross (Fig. 3d, e). Accordingly, we speculate that CmLEC1 may promote the synthesis of storage proteins, as well as oils and other key substances, by regulating the expression of those genes in chrysanthemum during embryo development, thereby finally increasing the seed-setting rate of chrysanthemums. Nevertheless, the enhanced expression of maturation genes in the OE-LEC1 cross may also be a read-out of the enhanced seed-setting rate^42^. LEC1 is an atypical subunit of the NF-Y CCAAT domain; it is a component of NF-Y complexes and may play the role of precursor TFs in different developmental processes^12,31,43,44^. LEC1 is a central transcriptional regulator of seed development, because it can govern different developmental processes at differing stages, including embryonic morphogenesis, photosynthesis, hormone biosynthesis, and signal transduction, as well as the large accumulation of seed storage macromolecules^15^. In our research, *CmLEC1*’s mRNA transcripts were mainly detected in the ovary and embryo, and its abundance of mRNA increased during normal chrysanthemum embryo development. We observed significantly higher expression levels in NE18 (normal embryo, 18 DAP) than in NE12 (normal embryo, 12 DAP); however, this greater expression was largely absent in AE18 (aborted embryo, 18 DAP). These results revealed a specific expression pattern of *CmLEC1* in the ovaries and normal embryos (Fig. 1d), suggesting its participation during the normal embryo development of chrysanthemums.

Previous studies have shown that CmLEC1 can interact with specific combinations of TFs to regulate specific gene sets during different developmental times in plants; for example, LEC1 and ABI3 work together to regulate the *OLE1* in Arabidopsis^14,45^. In this study, through the Y2H, LCI, and BiFC assays, it was revealed that CmLEC1 interacts with CmC3H (Fig. 5). In comparison with other DNA-binding proteins, there are many types of zinc finger proteins, and mounting evidence in recent years indicates that zinc finger proteins play an important role in plant growth and development^46–48^. Previous research has reported that the CCCH-type zinc finger protein, PEI1, is an embryo-specific TF that figures prominently in Arabidopsis embryogenesis, as it can bind to DNA and function as an embryo-specific TF operates that functions primarily in the apical domain of the embryo^49^. Similarly, overexpression of *BoC3H* contributes to seed germination in transgenic broccoli plants^50^. In some plants, C3H is an important driver of protein–protein interactions occurring at the endoplasmic reticulum (ER)^51,52^. For instance, subcellular localization of RgC3H to the ER was confirmed in *Rehmannia glutinosa*, but genetic manipulation showed that *RgC3H* positively promotes its release via molecular networks of the activated phenolic acid pathways^53^. Many zinc finger TFs display a variety of functions ranging from DNA or RNA binding to participating in protein–protein interactions. Hence, their activity is not limited to transcriptional regulation^54^, since one finds many proteins harboring a CCCH-type zinc finger motif that binds to RNA or DNA to perform their biological functions^55,56^. In our study, the mutual interoperability of CmC3H and CmLEC1 was confined to the nucleus, implicating its function as a TF. Comparative expression analysis of maize TFs associated with seed development revealed that ZmZF17, a zinc finger CCCH-type family protein, whose putative ortholog is At1g32360 in Arabidopsis, was preferentially expressed in the seed stage^57^. Similarity, in our study *CmC3H* was also specifically expressed in chrysanthemum embryos, matching a similar pattern found for *CmLEC1* in the normal developing embryos (Fig. 4e). Moreover, the interaction between CmLEC1 and CmC3H was only detected in the nucleus (Fig. 5c, d). Accordingly, these results implied the CmLEC1–CmC3H interaction might function in the nucleus by acting as a transcriptional complex.

LEA is a large type of hydrophilic protein, one associated with dehydration tolerance in plants during the embryos’ maturation^40,58,59^. Here we measured the expression of *CmLEA* by qRT-PCR, finding that *CmLEA* displayed higher transcript levels in the ♀OE-*CmLEC1*-*C.m*.×♂*C.n*. cross but lower levels in the ♀amiR-*CmLEC1*-*C.m*.×♂*C.n*. cross, when compared with the ♀*C.m*.×♂*C.n*. cross (Fig. 3e). This indicated that *CmLEA* could be regulated by CmLEC1. From the above analysis, we learned that CmLEC1 directly regulates the expression of *CmLEA* by associating with a CCAAT element upon its promoter (Fig. 6). As we all know, NF-YB participates in transcription regulation by binding to the CCAAT-box element in the promoter of the target gene^43^. Research has shown that LEC2 and LEC1 are partially co-localized in the nucleus of developing embryos, where the binding of LEC1 to the TFs containing B2 domain can form heteromers that are involved in the gene expression regulation^16,60^. We also observed that, when CmC3H was co-expressed with CmLEC1, this significantly enhanced the transactivation ability of CmLEC1 for the expression of *CmLEA*. This suggests that the CmLEC1–CmC3H combination might have a synergistic function in the expression of downstream genes.

**Fig. 7:**
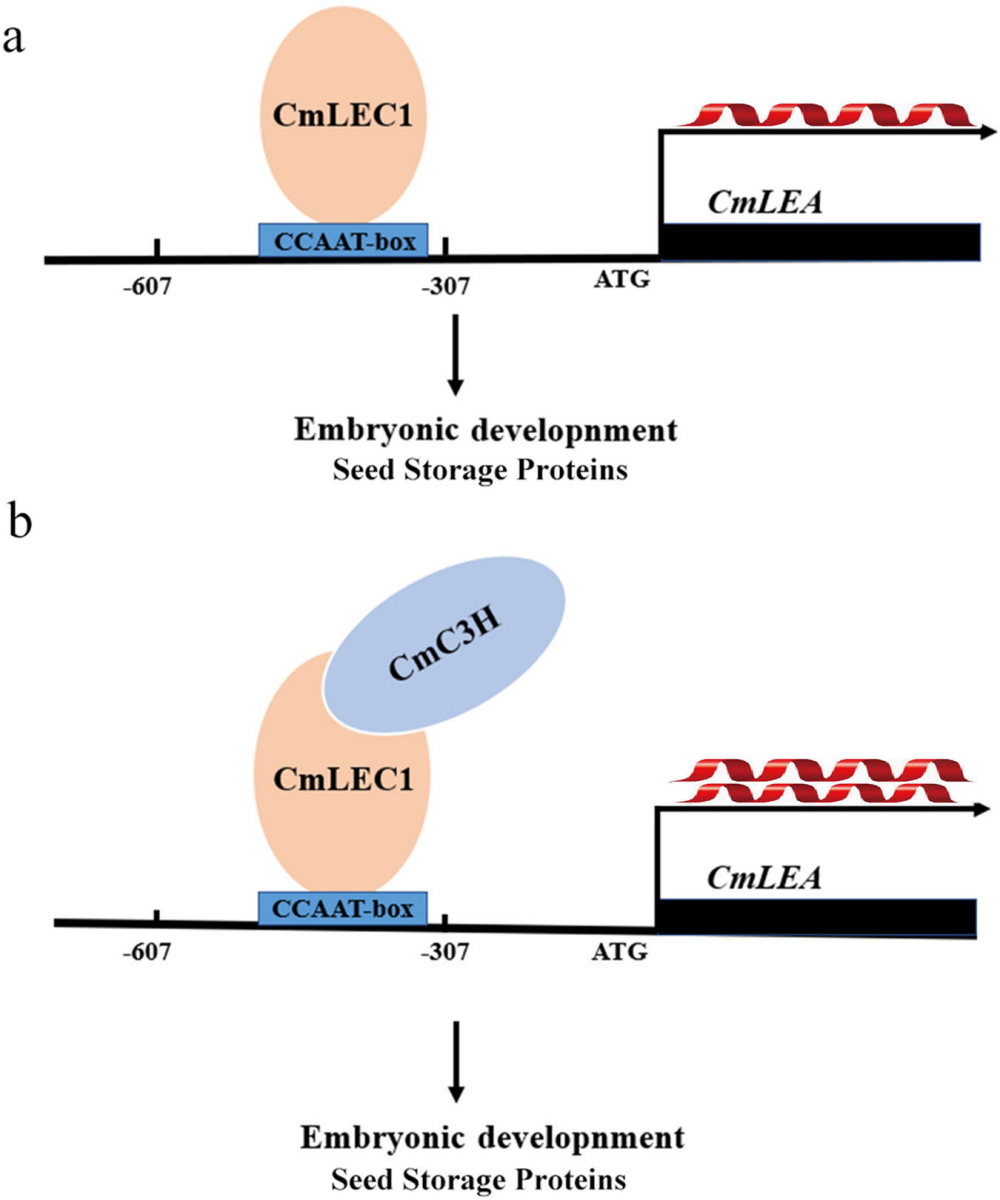
Working model of the CmLEC1-mediated regulatory mechanism of embryo development in chrysanthemum. CmLEC1 interacts with an embryo development factor, CmC3H, which together get bound to the upstream promoter region of *CmLEA* to positively promote the normal development of chrysanthemum embryos. (**a**) CmLEC1 gets bound to the upstream promoter region of CmLEA. (**b**) CmLEC1 interacts with an embryo development factor, CmC3H, which together get bound to the upstream promoter region of CmLEA

To conclude, we propose a possible working model for how CmLEC1 augments the seed-setting rate in the hybridization breeding of chrysanthemum plants (Fig. 7). Specifically, CmLEC1 interacts with a CCCH-type zinc finger protein factor, CmC3H, and this directly binds to the CCAAT element in the upstream promoter region of the *CmLEA* gene to positively promote chrysanthemum’s embryo development, consequently increasing the seed-setting rate. Put differently, CmLEC1 may promote the seed-setting rate by inducing *CmLEA* expression and strengthening it by interacting with CmC3H. Through this mechanism, LEC1 ensures that normal seed development unfolds, which could offer a possible, novel potential strategy to increase the seed-setting rate in hybridization breeding programs of crops.

## Material and methods

### Plant materials and artificial hybridization

In the study, the chrysanthemum materials used consisted of the cultivated ground-covering chrysanthemum ‘Yuhualuoying’ and a tetraploid species (*C. nankingense*), which are preserved in the Chrysanthemum Germplasm Resource Preserving Center (Nanjing Agricultural University, China). The tetraploid *C. nankingense* is an autotetraploid that was obtained by doubling the diploid *C. nankingense*, in a process induced by colchicine^61^. The transplanted transgenic line and WT of ‘Yuhualuoying’ chrysanthemums were used as female parents and the tetraploid *C. nankingense* as the male parent in an artificial hybridization. The interspecific hybridization for *C. morifolium* × tetraploid *C. nankingense* was performed according to our previous method^62^. To obtain robust statistics of the seed-setting rate, we planted >100 chrysanthemum individuals in total, and generated ca. 6000 inflorescences for the artificial hybridization. Meanwhile, the interspecific cross of *C. morifolium* × tetraploid *C. nankingense* was conducted with ca. 100 inflorescences to determine the seed-setting rate (by counting) at 2 months after pollination. The data were analyzed by SPSS version 20.0, and the average values between groups were compared by Student’s *t* test when alpha level = 0.05. Because chrysanthemum embryos are too small to be collected manually, and given that endosperm development also affects the embryo development, we instead sampled the developing ovaries after pollination for the gene expression analysis, as described in our prior study^25,63^. At 12 DAP, almost all the embryos had reached the spherical embryo stage, and the ovaries were collected under a dissecting microscope affixed with a digital camera. At 18 DAP, the full ovaries contained normal embryos (which reached the heart-shaped embryo stage), while the shrunken ovaries contained aborted embryos; these full and shrunken ovaries were separately collected.

### RNA extraction and qRT-PCR analysis

Total RNA was extracted from each chrysanthemum sample with the Trizol reagent, by following the manufacturer’s protocol (TaKaRa, Tokyo, Japan), and these isolated RNAs were stored at −80 °C until used in the qRT-PCR analysis. Single-stranded cDNA was obtained using the M-MLV Reverse Transcription Kit (TaKaRa) and qRT-PCR was implemented on a LightCycler^®^480 Real-Time PCR System. All qRT-PCR analyses were performed with three biological replicates. The primer pair *CmLEC1*-RT-F/R was used to amplify the fragment(s) and the *Elongation Factor 1*α (*CmEF1*α) gene (KF305681) was used as an internal control. The expression levels of all candidate genes were determined according to the 2^−ΔΔCt^ method^64^.

### Sequence alignment and phylogenetic tree of CmLEC1

Total RNA was extracted from each chrysanthemum sample according to the manufacturer’s protocol (TaKaRa). The primer pair *CmLEC1*-F/R (F: 5’-TGGGAATCAAACATAATGGAACG-3’, R: 5’-AACGAACTAGCGTCACAATCTCA) was designed to amplify the *CmLEC1* sequence fragment. The amino acid sequence alignment for *CmLEC1* was performed by the DNAMAN 5.2.2 software package and online, using the BLAST software (http://www.ncbi.nlm.gov/blast). Phylogenetic tree was constructed with MEGA 7.0^65^.

### Subcellular localization of CmLEC1 and CmC3H

The coding sequences (CDSs) of *CmLEC1* and *CmC3H* were inserted into the pCAMBIA1300-GFP vector to generate the recombinant expression vectors pCAMBIA1300-CmLEC1-GFP and pCAMBIA1300-CmC3H-GFP. Each construct was transformed into *A. tumefaciens* strain GV3101. Then the pCAMBIA1300-CmLEC1-GFP and 35S::D53-RFP or pCAMBIA1300-CmC3H-GFP and 35S::D53-RFP were transiently co-transformed into tobacco leaves, respectively, and 35S::D53-RFP construct indicated the localization of nuclei^66^. After 48 h, the fluorescence of GFP and red fluorescent protein (RFP) was captured by laser confocal microscope (LSM800, Zeiss, Germany).

### Transcriptional activation assay of CmC3H

The transcriptional activation of CmC3H was determined via the yeast system. Specifically, for these assays, the CDS of CmC3H was ligated to the pGBKT7 (Clontech) vector. The resulting constructs pDEST-GBKT7-CmC3H, pDEST-GBKT7 (i.e., the negative control), and pBD-GAL4 (i.e., the positive control) were separately introduced into the AH109 yeast stain according to the manufacturer’s protocol (Clontech). Those transformants containing pDEST-GBKT7-CmC3H or pBD-GAL4 or pDEST-GBKT7 were selected on SD/-Trp-His and SD/-Trp medium (SD, Synthetic Dropout Media). Then the selected clones were placed onto SD/-Trp-His medium and cultured at 30 °C for 2–3 days, prior to using them in the assay of X-α-galactosidase activity.

### Chrysanthemum transformation and generation of transgenic lines

In order to study the function of CmLEC1, the primer pairs (*CmLEC1*-F/*CmLEC1*-R) were used to amplify the ORF sequence of *CmLEC1* and introduced a recognition site for SalI and NotI (*CmLEC1*-SalI-F/-NotI-R). The resulting pENTR1A-*CmLEC1* constructs were digested by NsiI to construct the overexpression plasmid pMDC32-*CmLEC1*, as previously reported^67^. The vector of pMDC32 was controlled by CaMV 2× 35S promoter. The methods implemented to construct pMDC32-amiR-*CmLEC1* followed those already reported on elsewhere^68^. The amiRNA-containing precursor, obtained by amplifying miR319 template, was inserted into the pENTR1A; the primers can be found in Supplemental Table S1-3. The OE (pMDC32-*CmLEC1*) and amiR knockdown (pMDC32-amiR-*CmLEC1*) constructs were then each introduced into competent *A. tumefaciens* EHA105 cells. Next, the Agrobacterium was used for the genetic transformation chrysanthemum via vacuum infiltration of their stem internodes^69^. DNA was extracted from selected plants according to that manufacturer’s protocol using a plant genomic DNA rapid isolation kit (Sangon Biotech, Shanghai, China), and this was used as a template to verify the transgenic status of the plant using the primer pair 35S-F/*CmLEC1*-Test-R (Supplemental Table S1-3). Moreover, the putative transgenic plants were detected by qRT-PCR, using the primers *CmLEC1*-RT-F/R (Supplemental Table S1-4).

### Y2H assay

To further investigate the regulatory role of CmLEC1 in the embryo development of chrysanthemum, a yeast double-hybridization library of chrysanthemum ‘Yuhualuoying’ was built using Invitrogen. This library used the RNA of ovaries at 18 DAP and those at 12 DAP. The two-hybrid experiment was carried out to screen for those proteins interacting with pGBKT7-CmLEC1 according to the manufacturer’s description (Clontech). The paired bait and prey plasmids were co-transformed into the Y2H gold yeast strain. The growth of transformed yeast cells was tested on three media SD/-Leu-His-Ade-Trp, SD/-Leu-Trp, and SD/-Leu-His-Ade-Trp added to X-α-Gal, for stringent screening of plausible protein interactions.

### BiFC assay

A BiFC assay was conducted using *N. benthamiana* cells. The CDS of *CmC3H* lacking the stop codon was inserted into the pSPYNE173 and pSPYCE(M) vector with the primer pair (Supplementary Table S1-5). Conversely, the CDS of *CmLEC1* lacking the stop codon was cloned into the pSPYCE(M) and pSPYNE173 vector. Then *A. tumefaciens* GV3101 containing these vectors were co-injected into *N. benthamiana* leaves. After 48 h, the fluorescence signals of GFP were observed using a laser confocal microscope (LSM800, Zeiss, Germany). The experiment was repeated at least five times, using different batches of *N. benthamiana* plants each time.

### LCI assays

To investigate the in vivo interaction between CmC3H and CmLEC1 proteins, a LCI assay was carried out, as previously described^70^, by observing the fluorescence in the transformed cells. First, the CDSs of CmLEC1 and CmC3H were, respectively, fused to N- and C-terminus or the C- and N-terminus of the luciferase reporter gene. Then *A. tumefaciens* cells containing the cLUC-CmC3H and nLUC-CmLEC1 or cLUC-CmLEC1 and nLUC-CmC3H constructs were infiltrated into tobacco leaves together, for which the cLUC/nLUC, cLUC-CmLEC1/nLUC, and nLUC/cLUC-CmLEC1 pairs served as the negative controls. The LUC activity was determined 72 h later, using a charge-coupled device camera (Tanon 5200, China). The corresponding data were taken from four independent biological replicates, each consisting of three technical replicates.

### Pull-down assays

To produce GST-tagged protein, the CDS CmLEC1 was cloned into pGEX-4T-1 vector. Then the CDS of CmC3H was inserted into pET-32a expression vector for His-tagged fusion. The two proteins were individually expressed and purified from *Escherichia coli* BL21 (DE3). Next, the recombinant GST-CmLEC1 fusion protein and His-CmC3H fusion protein were mixed in equal volumes, incubated, and purified on a GST column. Finally, the resultant pellet fraction was detected via western blotting, using an anti-His antibody (Abmart).

### RNA-Seq and analysis

To obtain samples for the RNA-Seq, under a light microscope, we collected ovaries at 12 DAP from plants of the *C.m*.×♂*C.n*., the lan*CmLEC1*-*C.m*.×♂*C.n*., and the ♀ and*-CmLEC1*-*C.m*.×♂*C.n*. crosses. For each, two independent biological replicates were used. Total RNA was extracted using RNAiso Plus (TaKaRa) and following the manufacturer’s protocol. Then the mRNA was enriched by the addition of polyA tail after which cDNA was synthesized to construct the library, whose quality was checked and sequenced after passing. High-throughput sequencing was done on the BGISEQ-500 platform (BGI, Shenzhen, China) to yield 150 bp paired-end reads. In this study, the raw reads were imported into SOAPnuke v1.4.0 software to obtain their statistics, with Trimmomatic v0.36 then used to filter and obtain the clean reads, assembled with Trinity program reads. Next, the single-copy orthologous database BUSCO was relied on to evaluate the quality of assembled transcripts, after which Tgicl was used to cluster them according to their redundancy, to finally obtain the unigenes. TransDecoder software was used to identify coding region of each unigene. The assembled unigenes were functionally annotated by searching databases and RSEM was used to calculate the expression levels of each gene and its transcripts^71,72^. A *Q*-value < 0.05 was deemed the threshold for designating the DEGs^73,74^. KEGG enrichment analyses of the annotated DEGs were conducted on the BGI Interactive Reporting System (https://report.bgi.com/ps/login/login.html). In addition, processed RNA-seq data of 249 genes that were upregulated in the OE-*CmLEC1* lines but downregulated in the amiR-*CmLEC1* lines and 70 genes that were downregulated in the OE-*CmLEC1* lines but upregulated in the amiR-*CmLEC1* lines have been provided as Supplementary Data, which are named as Supplementary Tables S1-6 and S1-7, respectively.

### Yeast one-hybrid assay

The gene CDSs were cloned into the pB42AD vector, while the putative promoter sequences were amplified by PCR and cloned into the pLacZi vector. The plasmids were transformed into the yeast strain EGY48, respectively, after which the yeast cells were selected on plates without Ura and Trp. The ensuing positive clones were then cultured on the selective medium containing X-gal to strictly screen for possible interactions, according to the procedures of Matchmaker One-Hybrid System.

### Dual-luciferase assay

The promoter sequences of *CmLEA* and a mutated *proCmLEA* were inserted into the pGreenII 0800-LUC to generate two reporter constructs (*proCmLEA*-LUC and mutant-*proCmLEA*-LUC), while the CDSs of *CmLEC1* and *CmC3H* gene were cloned into the pGreenII 62-SK-GFP to generate two different effectors. These vectors were individually transformed into the *A. tumefaciens* strain GV3101. The concentration ratio of bacteria used for the injection was 1:10:10 for pGreenII 0800-LUC: pGreenII 62-SK: P19. Then the Agrobacteria strain GV3101 transformed with the above vectors were injected into the young leaves of tobacco. The LUC and REN activities were analyzed in three separate experiments, for which at least three biological replicates were measured in each assay.

## Supplementary information

SUPPLEMENTAL MATERIAL

SUPPLEMENTAL MATERIAL

## Data Availability

The raw data of the three samples was uploaded to the NCBI under the accession number PRJNA723709. All data supporting this research result can be obtained in the paper and within its Supplementary Materials published online.
