# Supplementary material for: The transcription factor CmLEC1 positively regulates the seed-setting rate in hybridization breeding of chrysanthemum
Source: Hortic Res. 2021 Aug 10;8:191. doi: 10.1038/s41438-021-00625-9 (PMC8355372; doi:10.1038/s41438-021-00625-9)
Supplement: Supplementary file 2 — SUPPLEMENTAL MATERIAL [file 41438_2021_625_MOESM2_ESM.docx]

**Fig. S1**

**

**

**Fig. S1 Phylogenetic analysis of CmLEC1 and 13 Arabidopsis NF-YBs.** AtNF-YB1 (AT2G38880), AtNF-YB2 (AT5G47640), AtNF-YB3 (AT4G14540), AtNF-YB4 (AT1G090300), AtNF-YB5 (AT2G47810), AtNF-YB6 (AT5G47670), AtNF-YB7 (AT2G13570), AtNF-YB8 (AT2G37060), AtNF-YB9 (AT1G21970), AtNF-YB10 (AT3G53340), AtNF-YB11 (AT2G27470), AtNF-YB12 (AT5G08190), AtNF-YB13 (AT5G23090). Bootstrap values indicate the divergence of each branch. Bar=0.1

**Fig. S2**


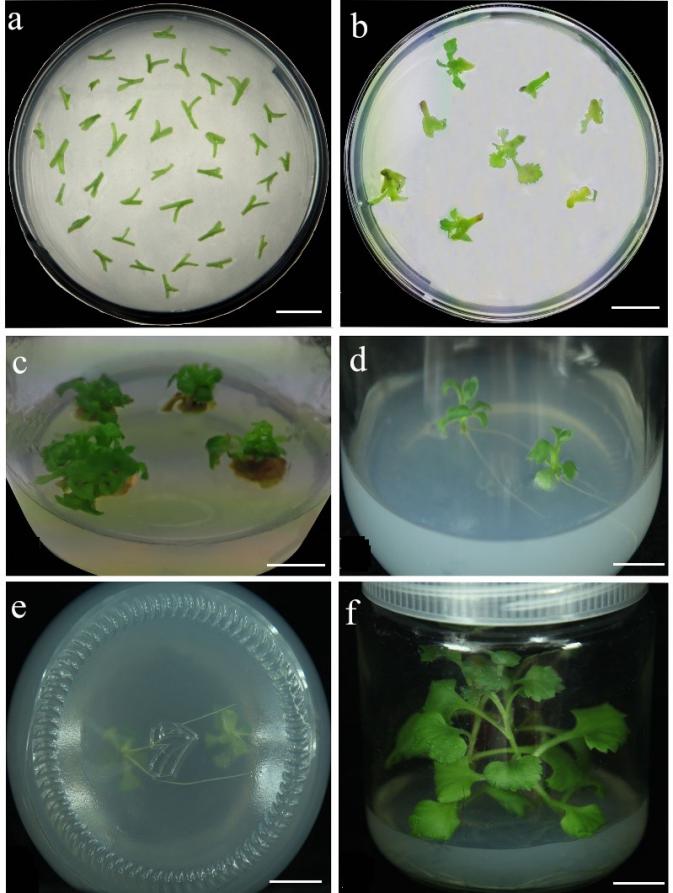


**Fig. S2 Chrysanthemum Transformation and Generation of Transgenic Lines. a** Pre-culture stage. **b** Decarboxylation stage. **c** Resistance screening stage. **d** Rooting screening. **e** Rooting stage root map. **f** Transgenic lines. Bars=10 mm.

**Fig. S3**

**
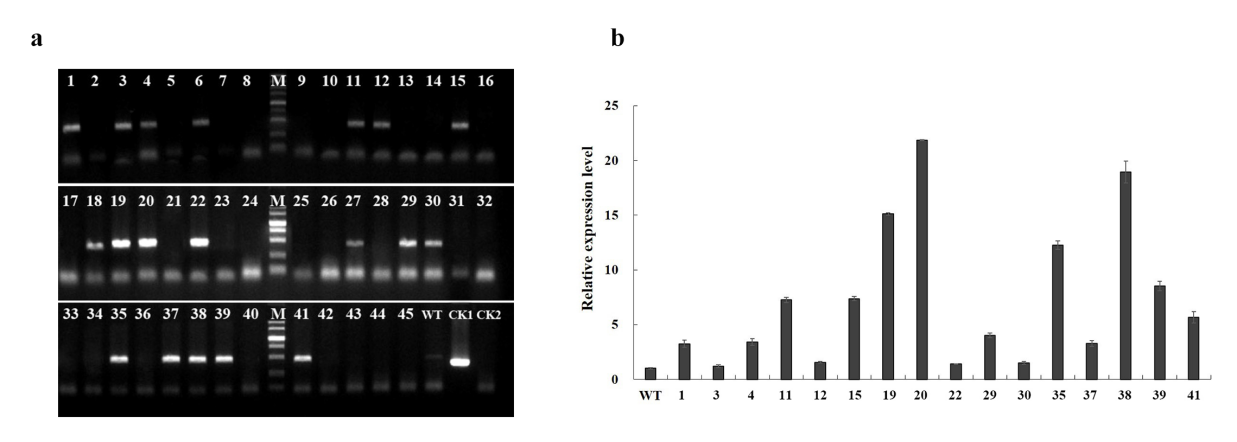
**

**Fig. S3** **Transgenic plantlet identification. a** The identification results of OE-*CmLEC1*transgenic seedlings based on a PCR assay of Genomic. M: molecular marker. CK1: Plasmid DNA (positive control). CK2: water (negative control). **b** A qRT-PCR assay quantifying the abundance of *CmLEC1* transcript in OE-*CmLEC1* plants. Values shown are means (n=3). Letters above the bars indicate significant differences as determined using a Student’s t-test. (P<0.05).

**Fig. S4**


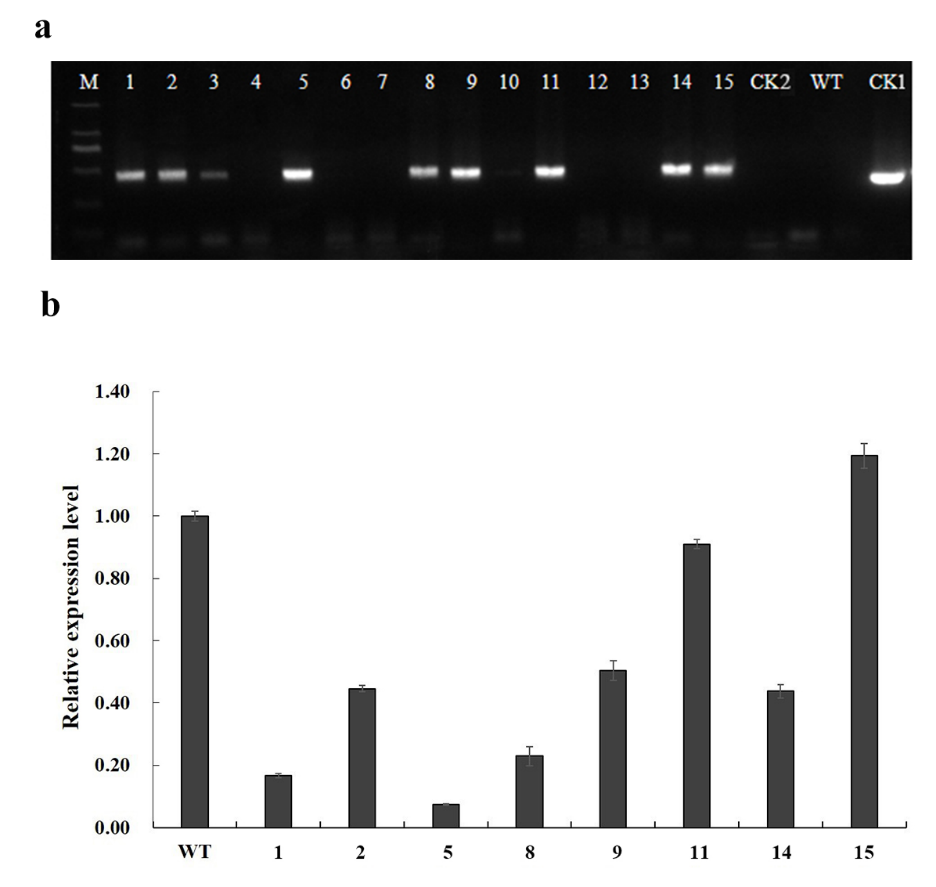


**Fig. S4** **Transgenic plantlet identification. a** The identification results of RNAi-*CmLEC1* transgenic seedlings based on a PCR assay of Genomic. M: molecular marker. CK1: Plasmid DNA (positive control). CK2: water (negative control). **b** A qRT-PCR assay quantifying the abundance of *CmLEC1* transcript in RNAi-*CmLEC1* plants. Values shown are means (n=3). Letters above the bars indicate significant differences as determined using a Student’s t-test (P<0.05).

**Fig. S5**

**
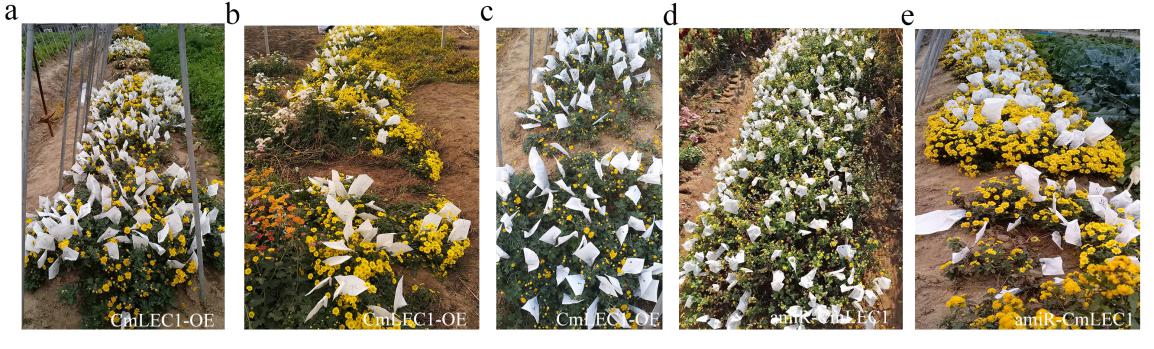
**

**Fig. S5 Hybridization process of transgenic *C. morifolium* ‘Yuhualuoying’ × tetraploid *C. nankingenese***

**Fig. S6**

**
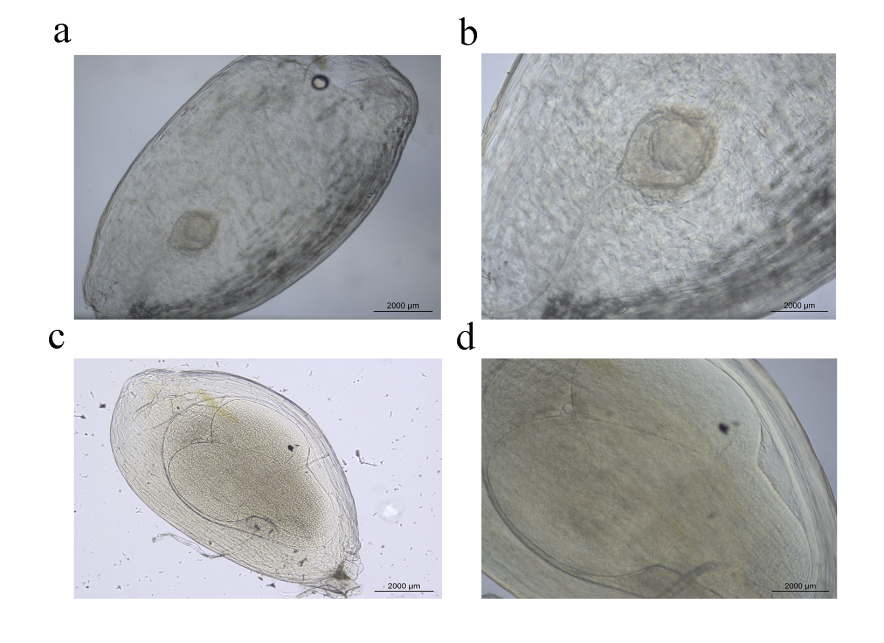
**

**Fig. S6 Morphological and anatomical features of chrysanthemum embryos**. **a b** Normal chrysanthemum embryo at 12 days after pollination (DAP). **c d** Normal chrysanthemum embryo at 25 days after pollination (DAP). Bar=2000 μm

**
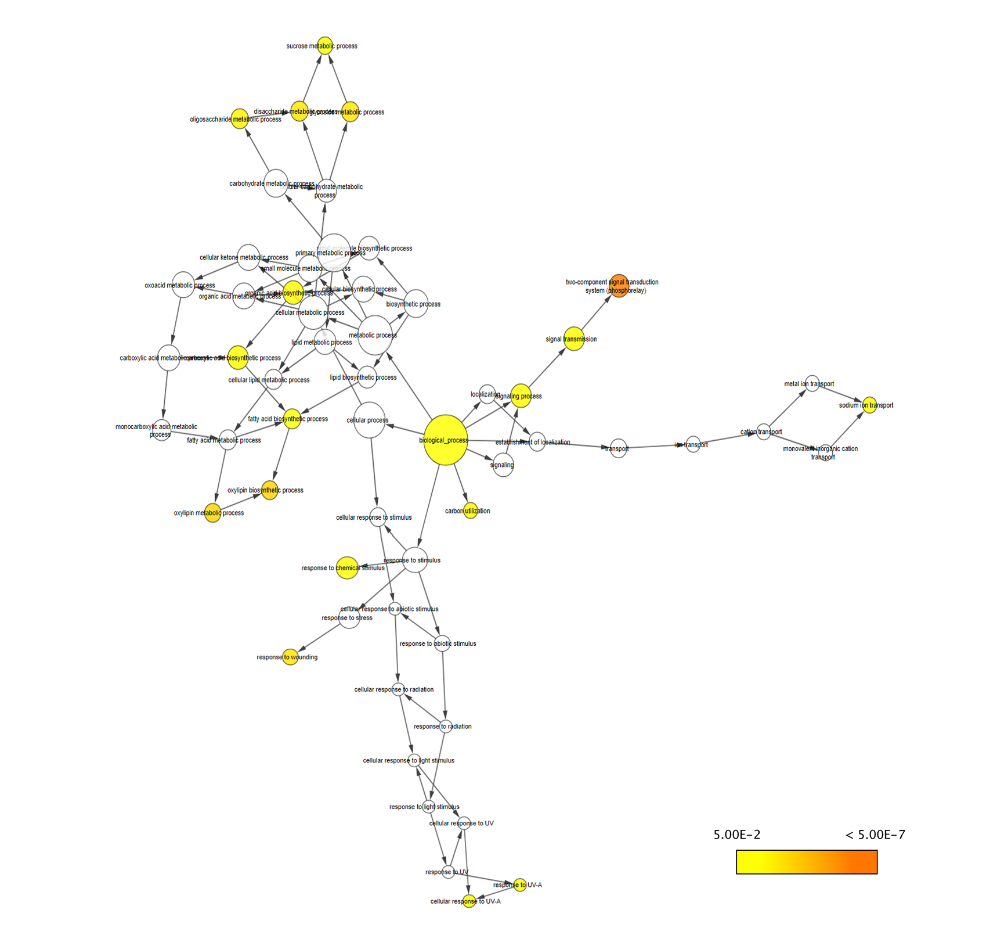
Fig. S7**

**Fig. S7 BinGO (Gene Ontology) functional analysis about the 249 genes that downregulated in the amiR lines but upregulated in the OE line.** The colour scale represents the p-value of enrichment of a category.

**
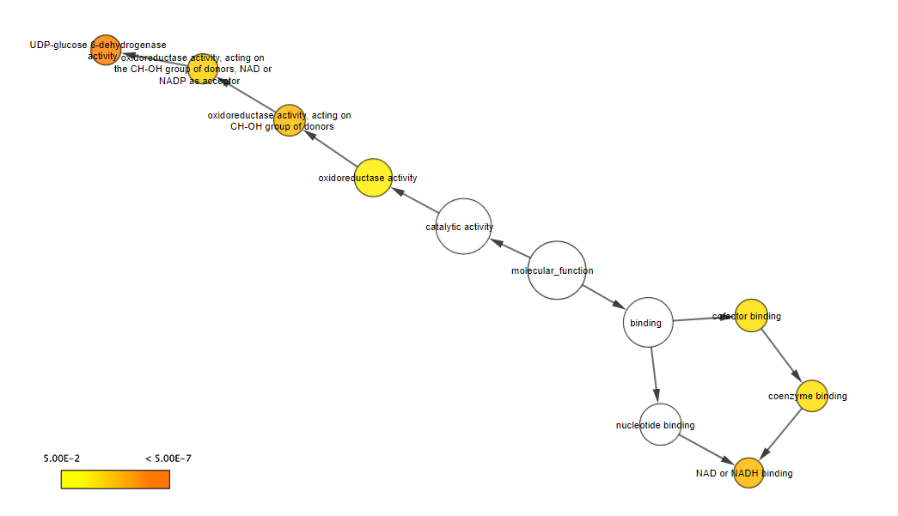
Fig. S8**

**Fig. S8 BinGO (Gene Ontology) functional analysis about the 70 genes that downregulated in the OE lines but upregulated in the amiR line.** The colour scale represents the p-value of enrichment of a category.

**Fig. S9**

**
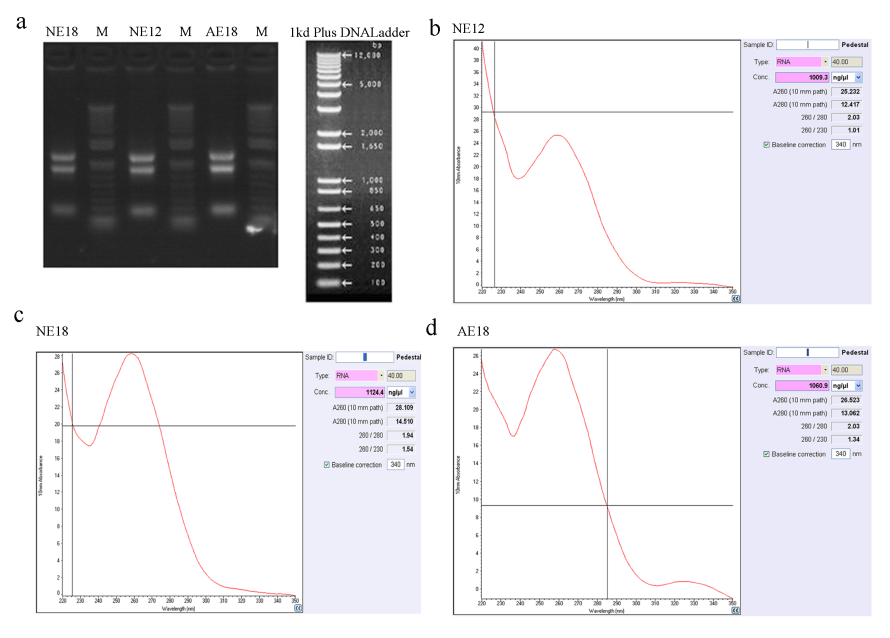
**

**Fig. S9 RNA extracted and RNA concentration and quality extracted from chrysanthemum embryos. a** Results of RNA extracted from chrysanthemum embryos. **b** RNA concentration and quality extracted from chrysanthemum embryos of NE12. **c** RNA concentration and quality extracted from chrysanthemum embryos of NE18. **d** RNA concentration and quality extracted from chrysanthemum embryos of AE18.

**Fig. S10**

**
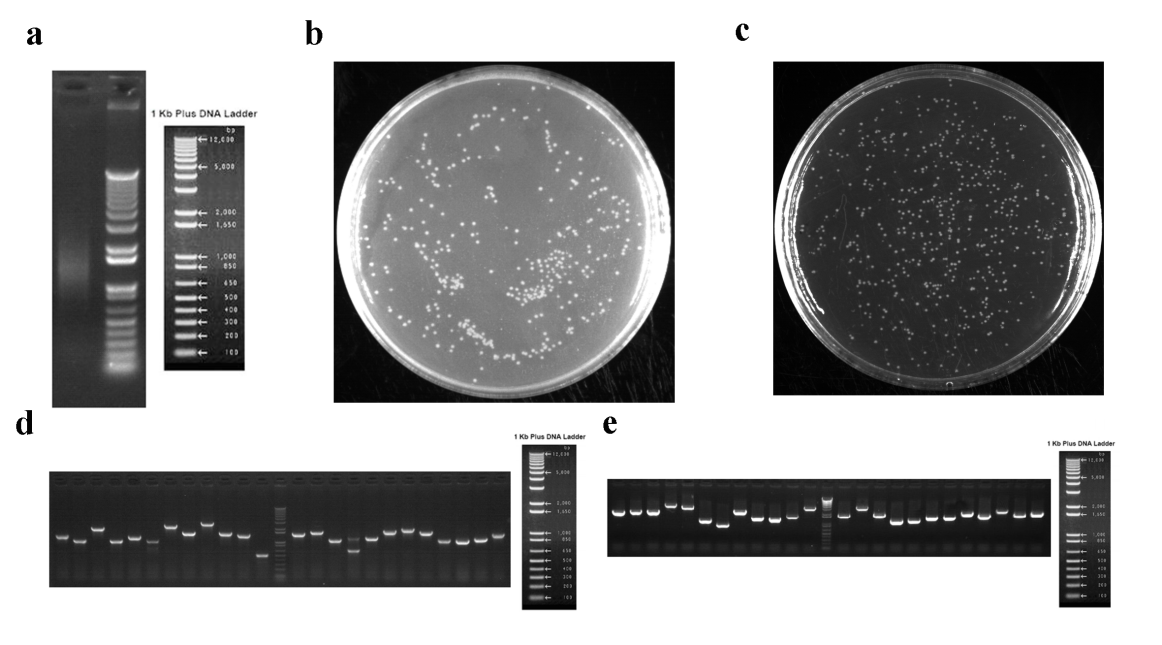
**

**Fig. S10 Construction of a yeast two-hybrid library. a** mRNA gel electrophoresis. **b** Primary library capacity identification. **c** Double hybrid secondary library capacity identification. **d** Primary library recombination rate and inserted fragments length identification. **e** Double hybrid secondary library recombination rate and inserted fragment length identification.

**Fig. S11**


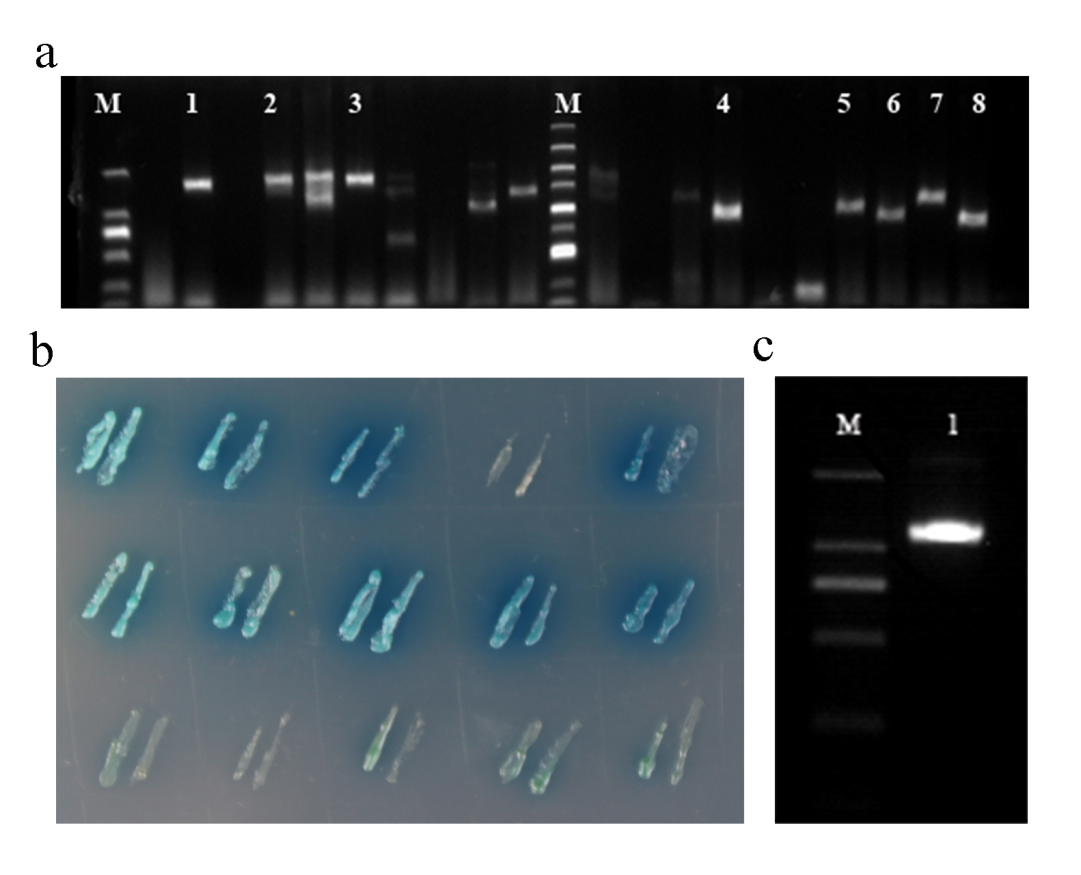


**Fig. S11 Yeast two-hybrid screening of interacting proteins and the PCR amplification result of *CmC3H.* a** Yeast identification by PCR. **b** Positive clone detection. **c** The PCR amplification result of *CmC3H* M: 2000 DNA Marker; 1: The ORF of *CmC3H*（ORF:1155).

**Fig. S12**

**
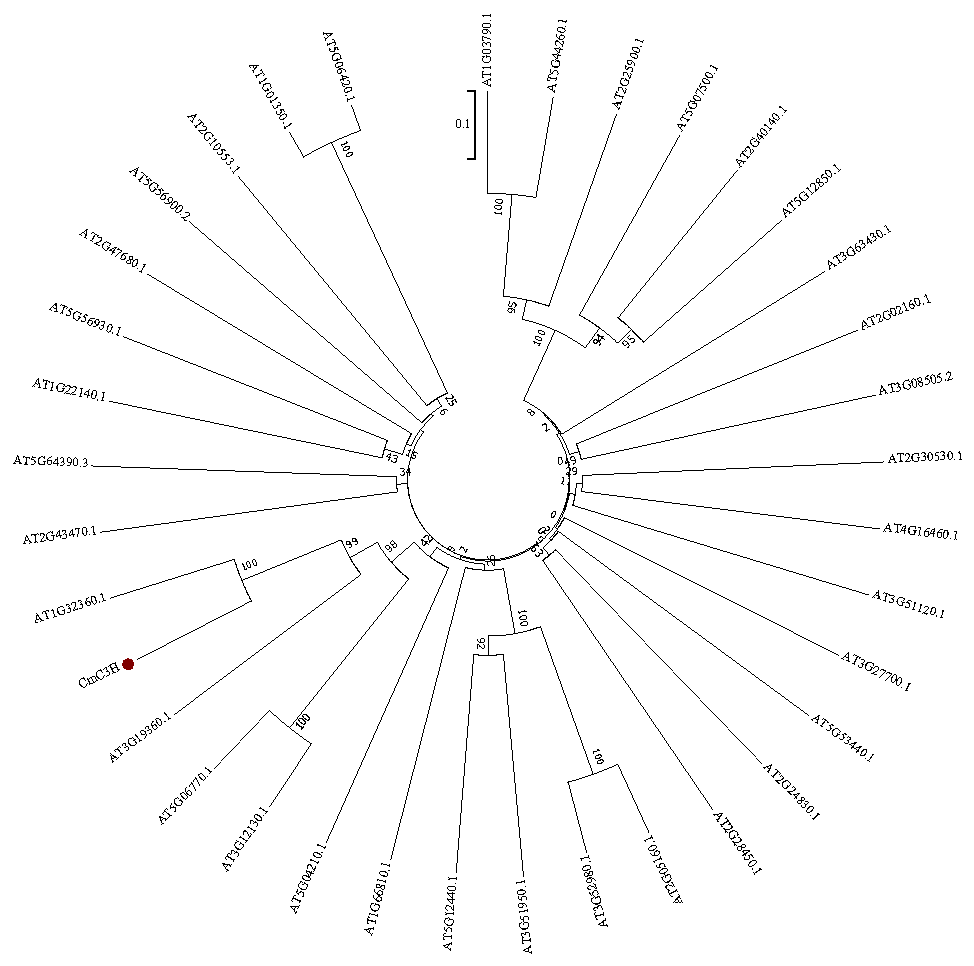
**

**Fig. S12 Phylogenetic analysis of CmC3H and Arabidopsis CCCH family**

**Fig. S13**

**
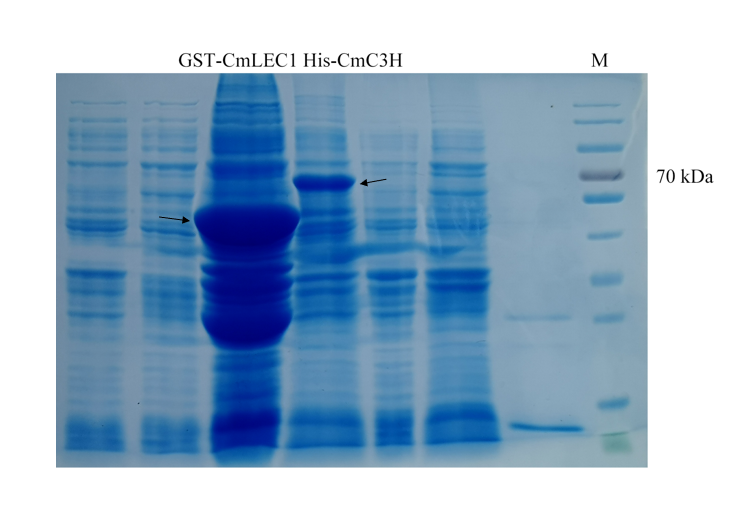
**

**Fig. S13 The test results of proteins molecular weight.** M: Protein Ladder. Recombinant CmC3H tagged with His (molecular weight of ~59 kD) and recombinant CmLEC1 tagged with GST (~51 kD) were successfully expressed and purified.

**Fig. S14**

**
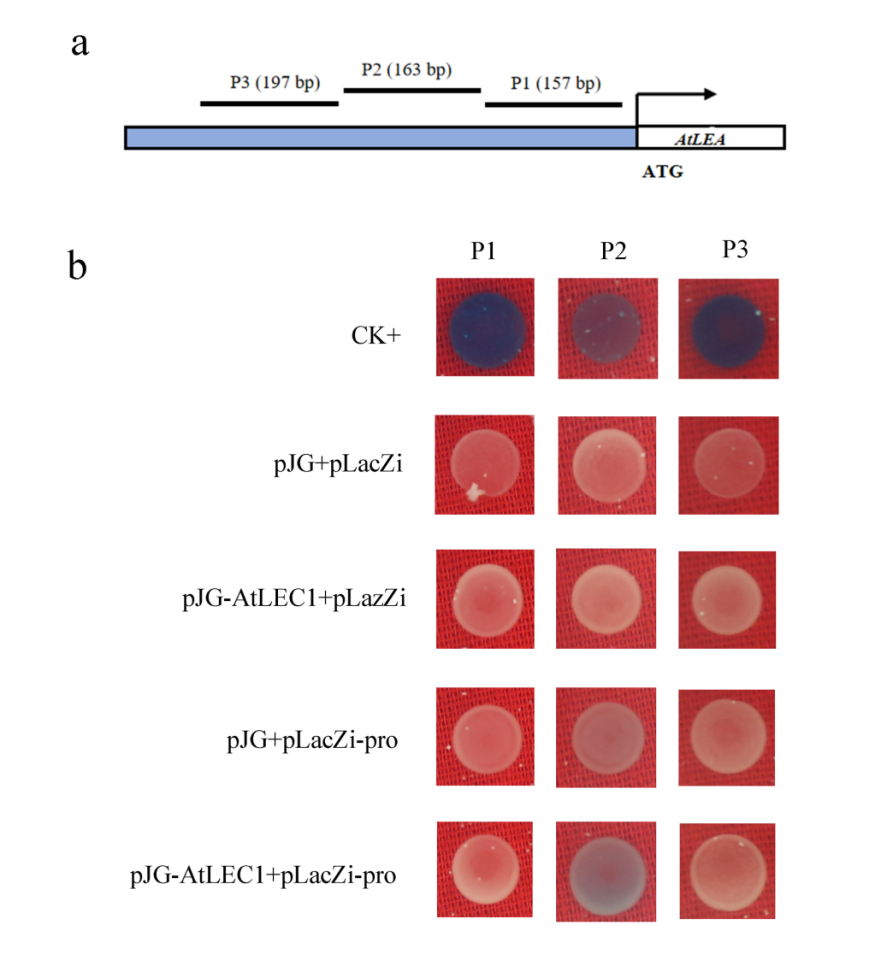
**

**Fig. S14 AtLEC1 binds to the promoter of *AtLEA.* a** Schematic representation of the upstream region of the foldback structure of the *AtLEA*. P1: 157 bp, P2: 163 bp, P3: 197 bp Lines below and above the blue box indicate the fragments used in the yeast one-hybrid analysis. **b** Analysis of AtLEC1 binding to the promoter of *AtLEA* in yeast one-hybrid system. The empty vector (pJG+pLazcZi) was used as a negative control. The promoters of *AtLEA* were ligated into the pLacZi vector, to drive the LacZ reporter gene expression in yeast cells, and the ORF of *AtLEC1* was cloned into pB42AD to generate pB42AD-AtLEC1.
